# Fast visual exploration of mass spectrometry images with interactive dynamic spectral similarity pseudocoloring

**DOI:** 10.1038/s41598-021-84049-4

**Published:** 2021-02-25

**Authors:** Karsten Wüllems, Annika Zurowietz, Martin Zurowietz, Roland Schneider, Hanna Bednarz, Karsten Niehaus, Tim W. Nattkemper

**Affiliations:** 1 International Research Training Group “Computational Methods for the Analysis of the Diversity and Dynamics of Genomes”, Bielefeld University, 33615 Bielefeld, Germany; 2 Biodata Mining Group, Faculty of Technology, Bielefeld University, 33615 Bielefeld, Germany; 3 Center for Biotechnology (CeBiTec), Bielefeld University, 33615 Bielefeld, Germany; 4 Proteome and Metabolome Research, Faculty of Biology, Bielefeld University, 33615 Bielefeld, Germany

**Keywords:** Data mining, Data processing, Image processing, Molecular imaging, Bioinformatics, Mass spectrometry, Metabolomics, Software, Software

## Abstract

Mass Spectrometry Imaging (MSI) is an established and still evolving technique for the spatial analysis of molecular co-location in biological samples. Nowadays, MSI is expanding into new domains such as clinical pathology. In order to increase the value of MSI data, software for visual analysis is required that is intuitive and technique independent. Here, we present QUIMBI (QUIck exploration tool for Multivariate BioImages) a new tool for the visual analysis of MSI data. QUIMBI is an interactive visual exploration tool that provides the user with a convenient and straightforward visual exploration of morphological and spectral features of MSI data. To improve the overall quality of MSI data by reducing non-tissue specific signals and to ensure optimal compatibility with QUIMBI, the tool is combined with the new pre-processing tool ProViM (Processing for Visualization and multivariate analysis of MSI Data), presented in this work. The features of the proposed visual analysis approach for MSI data analysis are demonstrated with two use cases. The results show that the use of ProViM and QUIMBI not only provides a new fast and intuitive visual analysis, but also allows the detection of new co-location patterns in MSI data that are difficult to find with other methods.

## Introduction

In clinical pathology, classical histology is still the most frequently used method for visual examination of tissue samples. Recently, digital histology emerged, moving histology to a new level regarding interpretation, analysis and research. Another complementing technology to analyze and classify tissue sections is the molecular histology by Mass Spectrometry Imaging (MSI). MSI is an emerging technique in the fields of metabolomics and proteomics, as it enables the identification of the spatial distribution of small molecules in tissue samples. In addition, the method can be combined with routine diagnostic analysis by immunohistological evaluation with antibodies. The classical analysis of histological staining requires a long learning phase and the results are difficult to classify for the untrained eye. In comparison, MSI measurements provide direct, label-free information about the underlying biological molecules, which usually cannot be detected by classical staining, such as metabolites, chemical or pharmaceutical compounds or lipids. But more importantly, interactive visual exploration of digital MSI data has the potential to become an efficient and effective method for tissue section interpretation with only little training as it can provide the additional level of molecular information in contrast to the classical histological staining.

For the visual exploration and analysis of MSI data two main dimensions have to be considered: the spatial (or lateral) dimension, which conveys information about the sample’s morphology, and the spectral dimension, which conveys information about the molecular composition, i.e. co-location at a pixel position. As it is impossible to show the entirety of all the information from both dimensions simultaneously in one static visualization, interactive visualization tools are required. With the additional goal to make MSI data more accessible for other domains, such as pathological, pharmaceutical or clinical research, using the visualization tool also needs to be intuitive for users with such a background.

Prior to an MSI data visualization, the data usually is subject to a (pre-)processing that addresses the special characteristics of multivariate bioimages such as of MSI^1^. This processing typically includes procedures such as signal alignment, signal normalization^2^, variance stabilization and peak picking. Another important step is the subtraction of signals that are not tissue-specific, e.g. matrix-related signals. In recent years, several tools for processing MSI data have been proposed. Some examples for freely available tools are SpectralAnalysis^3^, MSiReader^4^, BioMap (Novartis), Datcube Explorer^5^ and rMSI^6^, that focus on the interactive exploration of MSI data through basic mass channel visualization (such as pseudocolor maps). The R package Cardinal^7^ provides straightforward pre-processing of MSI data, dimensional reduction via principal component analysis (PCA) or partial least squares and clustering methods but does not provide an interactive visualization tool. The open source tool pyBASIS^8^ offers an extensive pre-processing pipeline with mass correction, normalization and variance stabilization transformation for large-scale sample sets and provides an automatic identification procedure of matrix peaks via *k*-means clustering. Commercial tools such as SCiLS (Bruker Daltonics), Xcalibur/ImageQuest (Thermo Fisher Scientific) and High Definition Imaging (HDI, Waters Corporation) provide straightforward pre-processing, a limited interactive visualization of mass channels and statistical methods such as PCA and *k*-means clustering.

Some of the aforementioned tools use methods such as dimensional reduction and clustering to automatically identify and exclude matrix pixels for further (statistical) analysis. And some of the tools also provide a transformation, e.g. logarithm or square root, to enhance weak signals and flatten strong signals. The first approach only excludes matrix pixels and the second one compresses the intensity range and is susceptible to the increase of noise signals. However, none of the tools allow a subtraction of tissue-unspecific mass signals in every pixel in a data set, which is often necessary to achieve a good signal contrast in the visualization. Using the identified matrix signal indices from pyBASIS, the user could subtract the matrix signals on their own. However, since this detection is fully automated, it does not provide the possibility to manually investigate and correct the result.

The visualization capabilities of all tools mentioned above are based on some kind of information reduction step. The tools show single mass channel images or the mean mass image computed from a selected range of mass channels. Some tools allow to restrict the visualization to a region of interest, updating the mass channel image or the mean image accordingly. Some tools offer other kinds of reduction based displays, such as a PCA projection.

Here we introduce a new method for visual MSI data exploration and analysis. This method combines the two new tools ProViM (Processing for Visualization and multivariate analysis of MSI Data) and QUIMBI (QUIck exploration tool for Multivariate BioImages) that are made available with this publication. ProViM is a processing method to prepare MSI data for the visual analysis, including the subtraction of tissue-unspecific signals (such as matrix signals) from the pixel mass spectra. The visualization tool QUIMBI is designed to support a fast and interactive exploration of processed MSI data that enables users to find new hidden regularities and co-location patterns in the MSI data without extensive training. The basic idea behind QUIMBI is to dynamically render pseudocolor maps for the MSI data set, that shows the (dis-)similarities of each pixels mass spectrum in relation to one freely chosen reference mass spectrum (selected in the pixel domain).

To demonstrate the usefulness of our method, we apply it to different kinds of MSI data. We compare different tissue sections of mice measured with MALDI-ToF (representing low-resolution MSI) to a MALDI orbitrap measurement (representing high resolution MSI). In addition, the visualization results from our method are also compared to classical staining using a tissue sample from human skin of a Pseudoxanthoma elasticum (PXE) patient. PXE is a rare disease of the connective tissue that leads to fragmentation of the elastic fibers and progressive calcification. Mainly affected are the Bruch’s membrane in the eyes, the skin and the arterial walls^9,10^. Because of the vascular affection, PXE may be a model disease for atherosclerosis. Our results show, that the combination of ProViM and QUIMBI can be flexibly applied to data sets obtained with different MSI techniques. Furthermore we show that the molecular distributions of tissue patterns that are visible in a classical staining (both in known and in unknown regions), can quickly be found and reproduced in QUIMBI by selection of the appropriate reference pixel. Most importantly, we demonstrate how QUIMBI can be applied to detect distributions of weak signal co-locations that are not visible through single or multiple mass channel visualization or in a classical staining.

## Results and discussion

**Figure 1:**
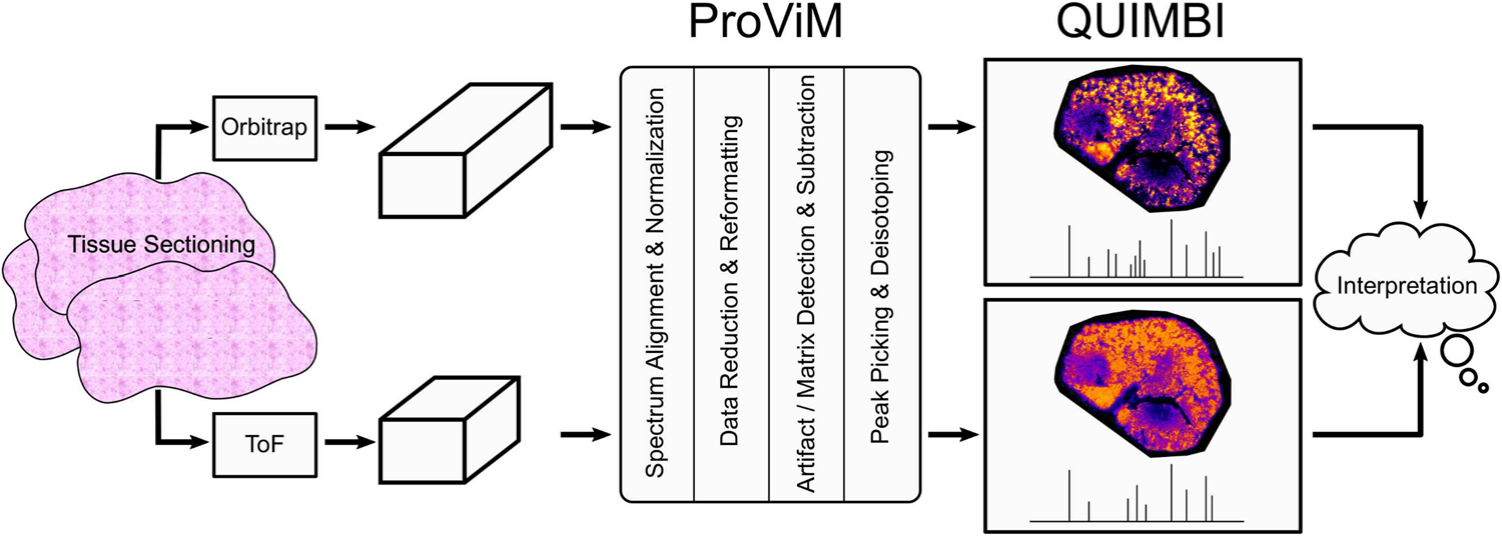
ProViM-QUIMBI workflow overview. After data set generation from either MALDI-ToF or MALDI-Orbitrap measurement ProViM successively executes four processing steps: (1) spectrum alignment and normalization, (2) data reduction and reformatting, (3) artifact and matrix detection and subtraction and (4) peak picking and deisotoping. Afterwards, the processed data set is interactively explored in QUIMBI.

The presented method for visual MSI analysis consists of two building blocks: (1) the processing tool to prepare the MSI data (ProViM) and (2) the interactive visualization tool (QUIMBI). While these two could be implemented as one in a monolithic manner, we chose to implement these into two separate software tools. This modular approach allows users, to apply both tools together consecutively (as we did in our studies presented here), or to combine one of the tools with another one from the realm of MSI software. For example, users might apply QUIMBI to MSI data that has been prepared with a different tool than ProViM. The whole workflow of ProViM and QUIMBI as proposed and applied in this work is illustrated in Fig. 1.

To demonstrate the usefulness of ProViM and QUIMBI, we used the following MSI data sets from four different tissue sections: (1) mouse kidney MALDI-ToF data (referred to as $I^\text {t}_\text {k}$), (2) mouse kidney MALDI-Orbitrap (consecutive tissue sections) data ($I^\text {O}_\text {k}$), (3) mouse vibrissae MALDI-Orbitrap data ($I^\text {O}_\text {v}$) and (4) human PXE skin MALDI-ToF data ($I^\text {t}_\text {s}$). For convenience, we will refer to an MSI data set with the variable *I* using the superscript to differentiate between instruments (t for MALDI-ToF and O for MALDI-Orbitrap) and the subscript to differentiate between samples (k, v, s for kidney, vibrissae and skin, respectively). In the following we describe the results of our method development and software implementation, as well as our two use cases. Algorithmic and technical details are given in “Material and methods” section.

The data sets have been processed with ProViM and visually explored with QUIMBI in two use cases. In the first use case, data sets (1)–(3) were analyzed to show that the molecular distribution pattern of a region of interest observed in a stained tissue section can also be found in QUIMBI by interactive exploration of the specific location on the image. The fourth data set was used in our second use case to demonstrate the ability of QUIMBI to reveal distributions that are neither visible to the untrained eye in the inspection of a sequence of single mass channels nor in the corresponding histological staining. The use cases show, that our methods can both reproduce known patterns and relationships in the MSI data and also detect new unknown patterns and regularities.

### ProViM

As mentioned above, a variety of different MSI pre-processing tools are already available. However, to prepare MSI data for a QUIMBI visualization we contribute our new lightweight software tool ProViM in this article. Different to other tools, it allows the interactive subtraction of non-tissue specific signals (such as matrix signals or artifacts) and an interactive peak picking, offering the user full control over these sensitive procedures. Although it is always advisable to use ProViM interactively, in situations where time is a limiting factor, e.g. in large series of experiments, it can also be executed fully automatically. Details about each pre-processing step applied in ProViM can be found in “Material and methods” section and in Supplementary A, C, D, E, J.

#### Matrix and artifact detection and subtraction

MSI pixels and their associated spectra can be classified into three categories: (A) sample signals, (B) matrix signals and (C) artifact signals. The categories A and B can be described as pixels that have a physical location inside the sample or in the matrix area. Category C includes pixels that can be located anywhere in the pixel grid showing mass spectra significantly different to the pixels mass spectra of categories A and B. Typically such artifacts appear as localized single intensity hotspots. These hotspots are unlikely to represent molecular distributions, therefore we refer to them as artifacts.

**Figure 2:**
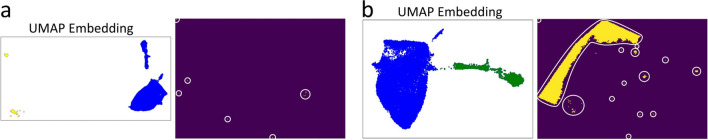
Interactive artifact and matrix subtraction in data set $I^\text {O}_\text {v}$ (mouse vibrissae section measured with MALDI-Orbitrap). (**a**) The UMAP embedding with clusters from sample and matrix signals (blue) and selected artifact clusters (yellow) are shown on the left. The corresponding artifact pixel positions are shown on the right and highlighted with white circles. (**b**) The UMAP embedding after removing artifacts. The sample cluster (blue) and the selected matrix cluster (green) are shown on the left. The corresponding matrix pixel positions are shown on the right and are highlighted with circles. The shown visualization is part of ProViMs interactive artifact and matrix subtraction module. The dimension reduction was computed by a successive combination of tSVD^11–13^ and UMAP^14,15^ as described in “Material and methods”.

The ProViM method to subtract signals of category B and C works in three steps: The first step calculates the embedding of the *d*-dimensional MSI data (with *d* being the number of $m/z$ values measured) into a two dimensional space applying UMAP (Uniform Manifold Approximation and Projection (for Dimension Reduction))^14^. In the second step, the artifacts and matrix signals are detected. This detection can be done automatically (see Supplementary J) or interactively, to have full control about this sensitive filtering step (see Supplementary B for details). In the third step, the mean spectrum of all pixel spectra that were classified as matrix is calculated and subtracted from the spectrum of all pixels of the data set. Users have the option to apply the same procedure to pixel spectra that were automatically (or manually) classified as artifacts. If strong artifact signals are present, this procedure can be applied in iteration. In the first step, the artifact pixels are removed. In the second step, the UMAP embedding is re-computed with the artifact-free data set. The re-computed embedding can show a more distinct separation between matrix and sample signals and is subsequently used to detect matrix pixels and to subtract the average matrix spectrum from each pixel of the data set. An example for the interactive classification is shown in Fig. 2 for the data set $I^\text {O}_\text {v}$. Further examples are given in Supplementary A.

In our experiments the ProViM matrix and artifact subtraction was performed independently for each data set. Matrix, such as the standard matrix 2,5-dihydroxybenzoic acid (DHB) used in our experiments, tends to show high and non-tissue specific signals. In our experiments, ProViM processing subtracted these dominant matrix signals which lead to substantially better peak picking results.

**Figure 3:**
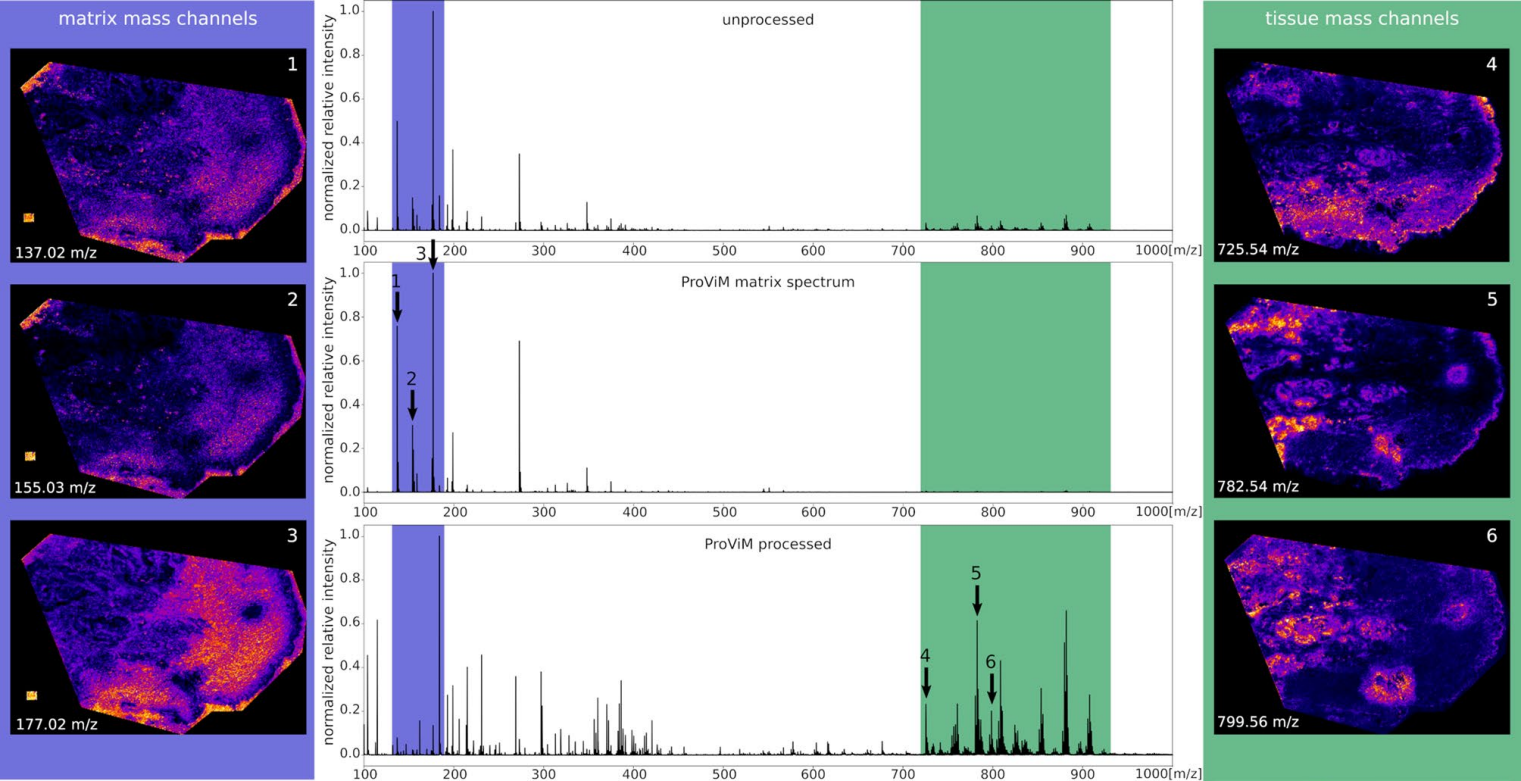
Result of the matrix signal subtraction step: Three average mass spectra normalized to [0,1] of the human PXE skin data set ($I^\text {t}_\text {s}$) measured with DHB matrix and MALDI-ToF are shown in the center column. The topmost mass spectrum shows the average spectrum before matrix subtraction. The middle MS is the approximate average spectrum of the matrix pixels manually selected in the matrix subtraction tool of ProViM. The main matrix mass range is highlighted with a blue box. Three representative mass channels are marked with arrows and their corresponding single mass channel images with matrix specific distributions are shown in the left column. The lowest MS shows the overall average spectrum after matrix subtraction with ProViM. The lipid region is highlighted with a green box and three representative mass channels are marked with arrows. The corresponding mass channel images with tissue-specific distributions are shown in the right column. ProViM matrix subtraction substantially enhances the mass signals of the lipid region of interest.

Figure 3 illustrates the effect of the matrix and artifact subtraction with ProViM. The figure shows three average mass spectra and corresponding single mass channel images of the human PXE skin data set $I^\text {t}_\text {s}$. The first mass spectrum (unprocessed) shows the average spectrum of $I^\text {t}_\text {s}$ before matrix subtraction. The second mass spectrum (ProViM matrix spectrum) shows the approximate average spectrum of the matrix pixels, manually selected in the matrix subtraction tool. The lower mass spectrum (ProViM processed) shows the overall average spectrum of $I^\text {t}_\text {s}$ after matrix subtraction. Figure 3 demonstrates that the high matrix signals (blue box) were greatly reduced after matrix subtraction with ProViM, resulting in tissue specific signals, e.g. the lipid region (green box), being much more pronounced.

To further demonstrate the advantage of matrix subtraction and its influence on the peak picking result, we have calculated the number of visible peaks before and after matrix subtraction with the same parameters for each data set. The number of peaks after matrix subtraction was 1.2 to 2.2 times higher when picked with exactly the same parameters (see Supplementary I). This indicates that the high outlier peaks coming from the matrix spectrum suppress many of the tissue-specific peaks. Without the matrix signal subtraction, the selection of a good picking threshold to separate the true tissue-specific signals from (weak) noise can be expected to be far more difficult.

In the course of this study and the presented results we did not observe that the ProVim matrix subtraction had an unwanted spoiling or disturbing effect on the resulting mass spectra. But it must be noted that such an effect can be ruled out in any way. There is no guarantee that the matrix subtraction procedure will not introduce artificial signals (bias) or reduce signals that are not related to matrix or artifacts. However, the overall benefit of reducing the matrix influence by subtraction in ProViM appears to be considerably greater than potentially inserted bias. Additionally, the interactive matrix subtraction gives visual control over the pixels declared as matrix, ensuring that no mass spectra of tissue pixels are involved in the subtraction. And in case of a detailed in-depth analysis of some pixel spectra following the visual exploration, users can always “go back” to the original unprocessed data.

The matrix subtraction procedure had a greatly positive impact on the quality of the QUIMBI visualizations. This is consistent with previous observations for dimension reduction-based visualizations^16^.

### QUIMBI

**Figure 4:**
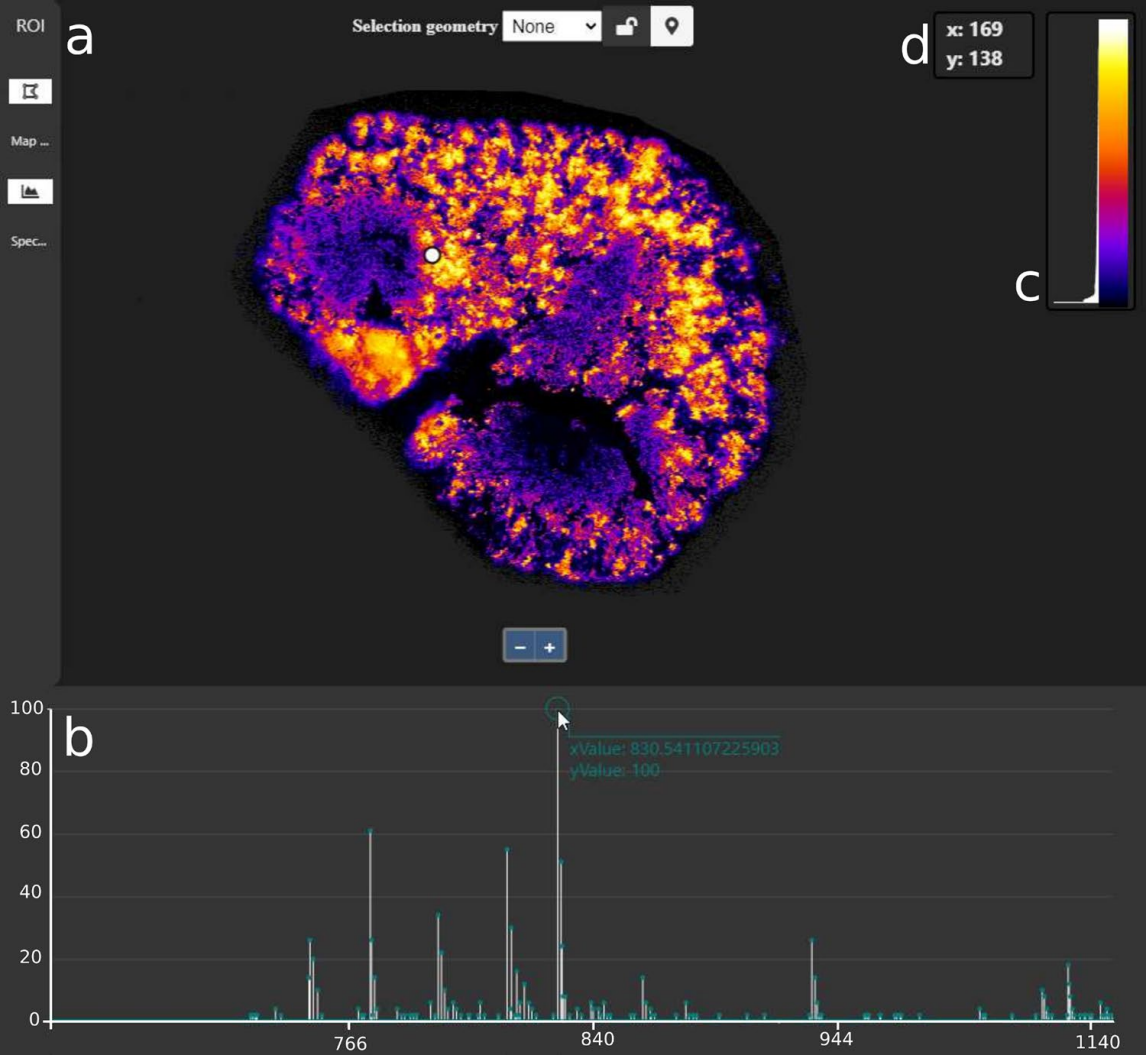
QUIMBI user interface. (**a**) The *main panel* with a selected reference pixel. The pseudocoloring shows the similarity of each pixel spectrum to the selected reference pixel. (**b**) The *spectrum viewer* shows the mass spectrum of the selected reference pixel. Hovering the spectrum with the mouse shows an annotation about the $m/z$ value and the intensity at the mouse position. (**c**) Color scale legend combined with a similarity value histogram plot. (**d**) Position indicator.

QUIMBI visualizes similarity regarding local molecular composition (or molecular co-location) within the morphological space of the sample (see Fig. 4). By selecting one reference pixel position (*q*, *r*) (see the white circle in Fig. 4), the pairwise spectral similarity $c'_{i,j}$ between the mass spectrum of the reference pixel $p_{q,r}$ and the mass spectrum of each other pixel $p_{i,j}$ is computed. According to these similarity values, every pixel position is colored using a pseudocolor scale. Each time when a new reference pixel is selected, the pseudocolors of all other pixel positions are updated according to the new similarity values. This dynamically changing pseudocolor map representation of the MSI data (e.g. Fig. 4a) enables an instant perception of the relation between the molecular composition of spectra (or molecular co-location) and the sample’s morphology^17^. An interactive example of QUIMBI, as well as instructions for the installation to enable the interactive exploration of the examples presented in this manuscript, can be found at https://github.com/BiodataMiningGroup/quimbi. Due to storage space restrictions and to provide a smooth running example even for low-end computers, we decided to use the small MSI data set of a barley germinated seed sample^18^ for the live demo.

Since QUIMBI is implemented in WebGL the application scales well with the lateral resolution due to the parallelization features of the GPU. The only limiting factors are the GPU speed, GPU memory and CPU memory. However, some limitation comes with using WebGL. The implementation requires a transformation of the intensity values to 8-bit precision, which means that the theoretical maximum intensity resolution per spectrum is limited to 256 different values. Technical details on how this is handled can be found in Supplementary K. This step reduces the data dimension considerably and naturally leads to the loss of details, like other data reduction strategies. This loss can be considered a potential source of false interpretations based on distorted visual mappings. To address the above concerns, we present a comparison of QUIMBI visualizations based on 32-bit representations, which for our data equals its original precision, versus the 8-bit representation in Supplementary K that show no strong misleading structural differences in the visualizations.

Another limitation is the spectral resolution, i.e. the number of $m/z$ values *d*. The higher the spectral resolution, the more computation time per GPU core is required to compute the similarity values. The similarity computation for a single pixel pair is not parallelized, meaning that it does not scale with the number of cores but with the power of each individual core. The trade-off between resolution and computation time becomes apparent by comparing the smoothness of a MALDI-ToF and a MALDI-Orbitrap data exploration, showing that the latter is noticeably slower. However, although the resolution of the MALDI-Orbitrap data is already very high considering current MALDI techniques, a data set without peak picking with a mass channel sample range of $d=70{,}000$ values can be loaded and QUIMBI still runs smoothly enough for an efficient interactive exploration.

### Use case 1: QUIMBI shows molecular distributions of interest and reveals similar regions

**Figure 5:**
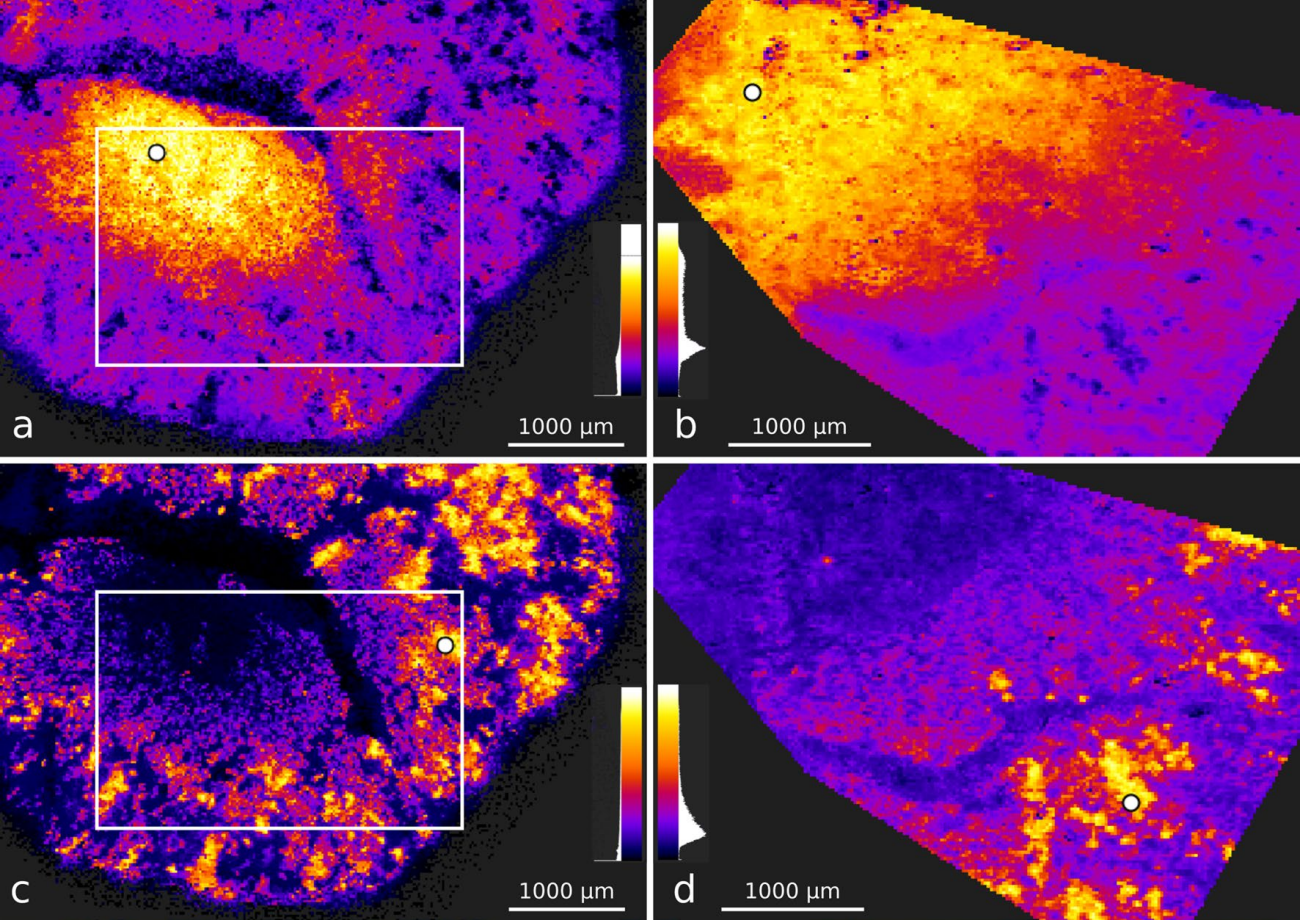
QUIMBI visualization of the mouse kidney data sets $I^\text {O}_\text {k}$ and $I^\text {t}_\text {k}$. $I^\text {O}_\text {k}$ (**a**,**c**) and $I^\text {t}_\text {k}$ (**b**,**d**) show similar spatial distributions in the visualization. The white circles mark the selected positions of the reference pixel. The white rectangles in (**a**) and (**c**) mark the corresponding region of the $I^\text {t}_\text {k}$ measurement in (**b**) and (**d**), respectively.

The QUIMBI visualization of the mouse kidney data sets (see Fig. 5) show a similar distribution of molecules both for MALDI-ToF ($I^\text {t}_\text {k}$) and MALDI-Orbitrap ($I^\text {O}_\text {k}$). The comparison of two example distributions is shown in Fig. 5a–d, whereby the same positions of the reference pixel are selected. Figure  5a,b show a molecular co-location in the renal medulla. Figure 5c,d show a molecular co-location in the renal cortex. Those distributions are obtained by selecting the reference pixel indicated with a white dot. A list of the ten masses with highest signals of these distributions as well as an exemplary classical staining of a consecutive tissue section is given in Supplementary F. By iteratively selecting different reference pixel positions directly on the tissue section display (see the white dots in Fig. 5), $I^\text {t}_\text {k}$ and $I^\text {O}_\text {k}$ were explored to quickly find and compare different co-location distributions. Furthermore, the mass spectrum of the selected reference pixel is displayed in QUIMBI, which makes it easy to obtain all mass channels that contribute to a certain distribution pattern.

**Figure 6:**
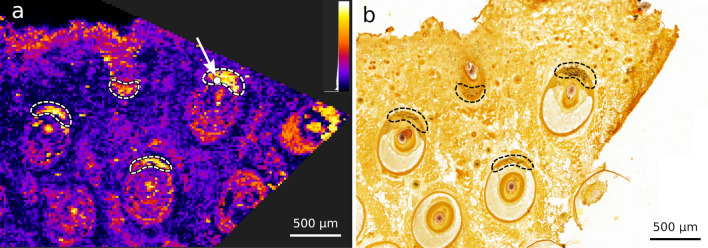
QUIMBI visualization of the mouse vibrissae data sets $I^\text {O}_\text {v}$ (**a**) with corresponding histology (**b**). The selected position of the reference pixel (white circle in (**a**)) is marked with an arrow. $I^\text {O}_\text {v}$ shows clear patterns at the outer edges of the vibrissae and also reveals the hair follicles as molecularly similar regions.

Figure 6a shows a QUIMBI visualization of the MALDI-Orbitrap data from the mouse vibrissae section $I^\text {O}_\text {v}$ that reveals distinct distribution patterns of similar pixels at the outer edges of the vibrissae (outlined with dashed lines). The histological staining of the mouse vibrissae section (Fig. 6b) shows clear structural features, i.e. distinct tissue categories can be clearly separated by different colors, textures and edges such as the capsule (outer circle) and the hair follicle (inner circle). In addition, the marked regions in the QUIMBI visualization are also visible as black spots in the staining (see Fig. 6b). The nature of these regions is unknown to the authors. The QUIMBI visualization shows not only unknown regions, which are visible in the staining, but also regions corresponding to similar molecular co-location. In this case the regions seem to be similar to the hair follicles, which are also brightly colored in the QUIMBI visualisation. Thus, in QUIMBI the co-location-based distribution patterns of (unknown) regions visible in a staining were found and the responsible mass channels can be identified. In other pathology research projects this kind of analysis could be useful if an unknown region has a similar mass distribution to a known region. In clinical pathology, a known distribution can be quickly identified and similar regions to this region are instantly shown. This could be useful, for example, with regard to tumor tissue, where the main tumor region can be quickly identified by a pathologist and with the help of QUIMBI all molecularly similar regions (in this theoretical case these could be metastasis cells) can be instantly detected without further analysis.

### Use case 2: QUIMBI reveals new distribution patterns

Another potential application of QUIMBI is to complement the interpretation based on classical staining, i.e. the identification of structural features in a sample that have not been visualized with classical staining. To demonstrate this use case, we visually explored the MALDI-ToF MSI data from selected skin sections of a PXE patient ($I^\text {t}_\text {s}$) and compared the results with the subsequent tissue section stained with Hematoxylin and Eosin (HE).

**Figure 7:**
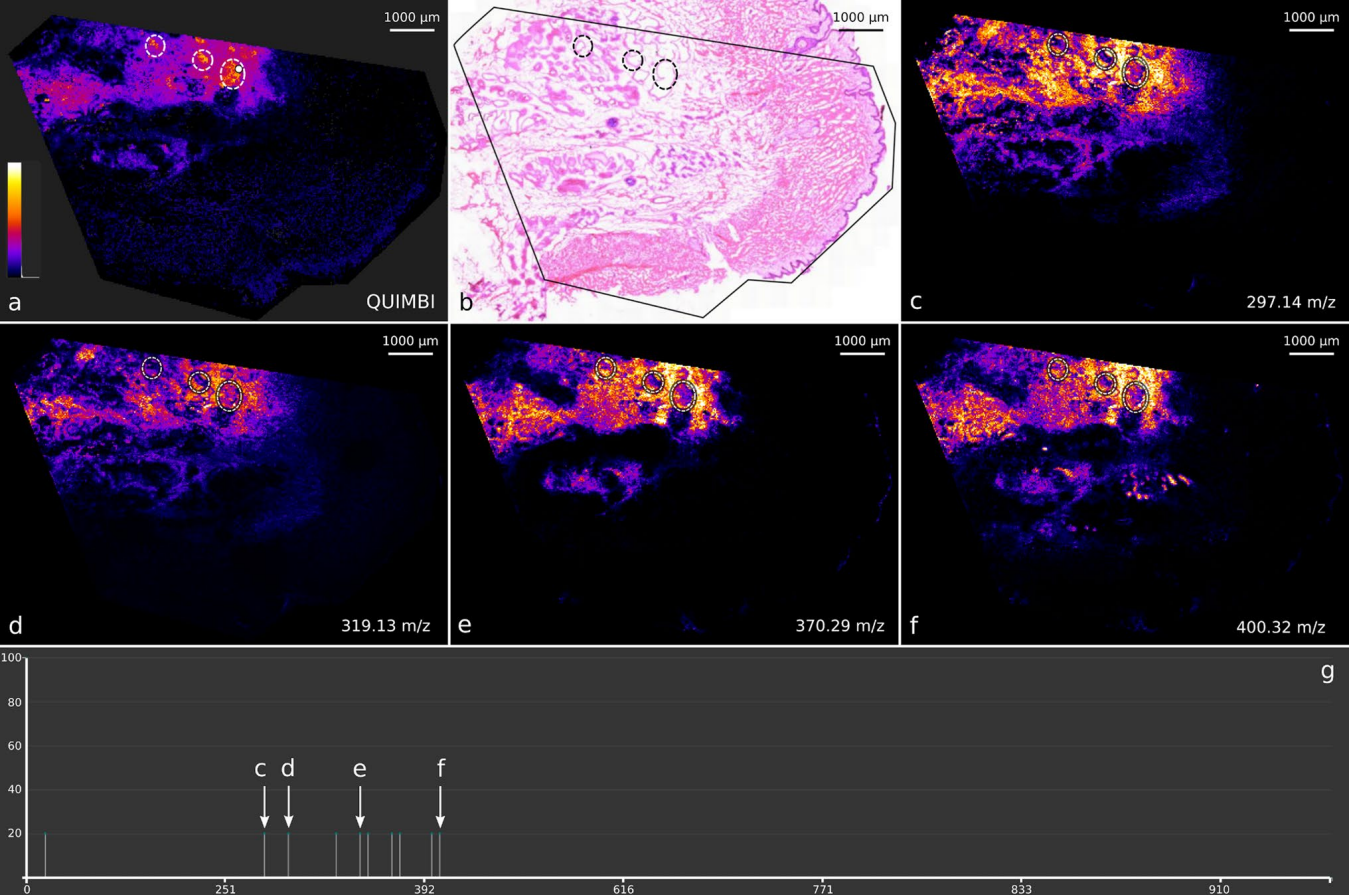
QUIMBI visualization (**a**) of human PXE skin showing a distinct distribution which is based on weak signals. (**b**) shows the corresponding histology and (**c**)–(**f**) selected single mass channel images. In this example visualization, the selected reference pixel in (**a**) comprises ten mass channels with a non-zero signal which are scaled to the same signal intensity due to the 8-bit scaling (**g**). The marked regions (dashed white lines) show a molecular distribution which is visible in the QUIMBI visualization (**a**) but not in the single mass channel images (**c**) to (**f**). All single mass channel images can be found in Supplementary G. The image intensity scale in (**a**) shows the similarity of pixels from black (dissimilar) to white (similar).

Figure 7 shows the QUIMBI visualizations of the skin PXE section $I^\text {t}_\text {s}$ together with a consecutive HE stained tissue section. Figure 7a shows the QUIMBI visualization of one selected reference pixel. Choosing this position revealed a specific localization pattern with a high contrast to the rest of the tissue section. Figure 7b shows the corresponding HE staining of the subsequent tissue section with no pattern such as shown in Fig. 7a visible. In Fig. 7c–f images of selected single mass channels are shown as an example. A figure with the images of all single mass channels displayed in the mass spectrum of the reference pixel in the QUIMBI visualization (see Fig. 7g) and the corresponding mean image is provided in Supplementary G. Although the patterns in Fig. 7c–f do overlap with the pattern in Fig. 7a to some extent, they also show some variations. Most importantly, none of the single mass channel images shows the same distribution pattern as the QUIMBI visualization, nor does it highlight the marked regions.

**Figure 8:**
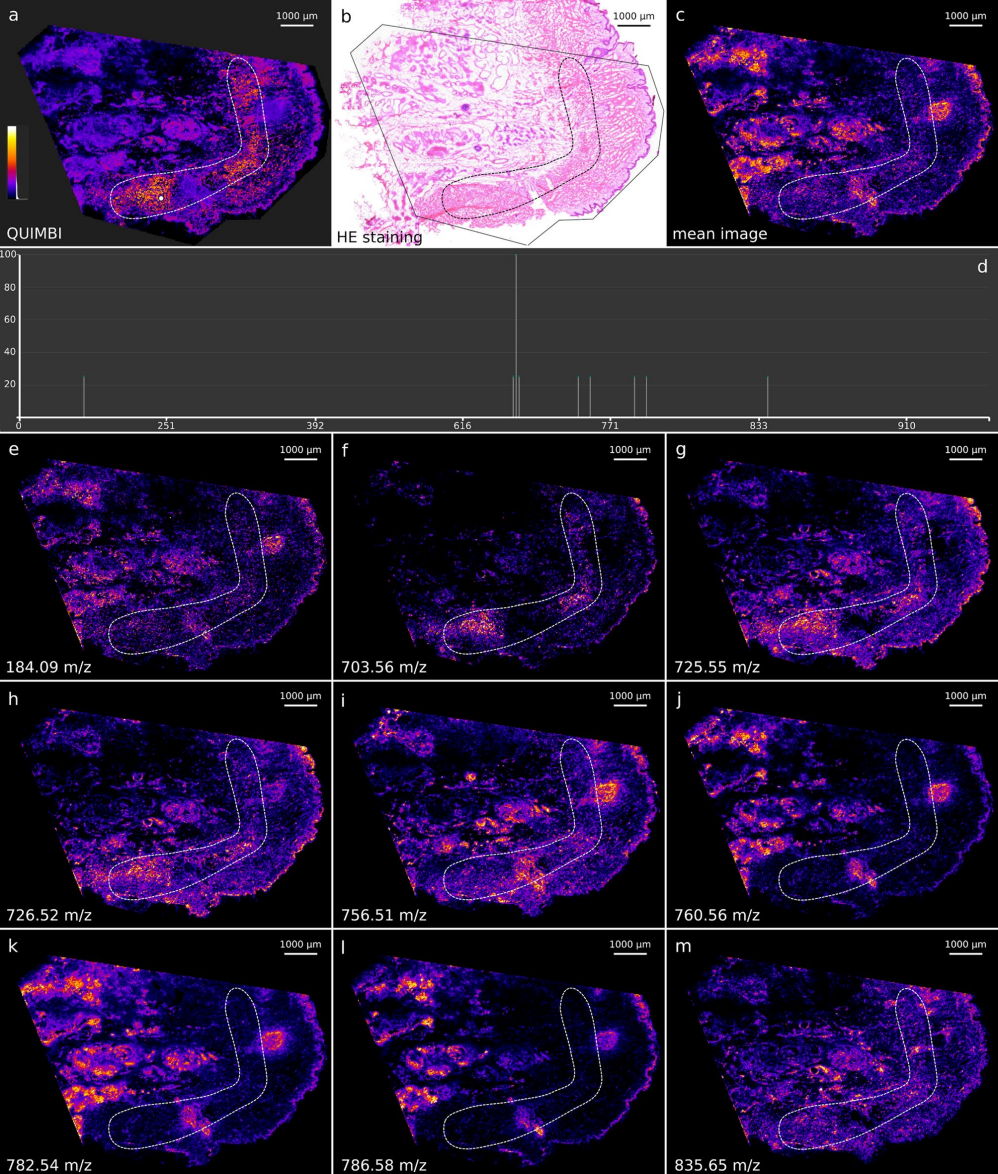
QUIMBI visualization of human PXE skin showing a calcification layer (**a**), with corresponding histology (**b**), mean image of all mass channels (**c**) and single mass channel images (**e**)–(**m**). The image intensity scale in (**a**) shows the similarity of pixels from black (dissimilar) to white (similar). The QUIMBI visualization in (**a**) shows a distribution of weak signals for the selected reference pixel (white circle in **a**). The dashed line in the QUIMBI visualization (**a**) marks a calcification layer in the skin that is characteristic for PXE. In (**d**) the mass spectrum belonging to the reference pixel selected in (**a**) can be seen, which shows nine mass channels with non-zero signal. In (**e**)–(**m**) the single mass channel images are shown. The mean image of the nine mass channels is shown in (**c**). In all images, the region shown in (**a**) is marked with a dashed line. Only the QUIMBI visualization fully reveals the calcification layer.

A larger pattern is shown in Fig. 8a. This region shows no contrast in the HE stain (Fig. 8b) although it is visually evident that it describes a border area between two morphologically different regions.

An alizarin stained human PXE skin section (see Supplementary G, Figure 7) reveals, that the region marked with a dashed line in Fig. 8a highlights a calcification layer of the skin sample that is characteristic of PXE. The corresponding mass spectrum of the QUIMBI visualization (see Fig. 8d) shows nine mass channels contributing to the visualization in Fig. 8a). When looking at the single mass channel images of these nine mass channels (see Fig. 8e–m), none of the images shows the entire calcification layer. Even a mean image of these nine mass channel images does not reveal the calcification layer (see Fig. 8c). Thus, the region of the MSI data set is described by the special co-location pattern of the chosen reference pixel (or by another reference pixel from the same region shown with high values in Fig. 8a) and only revealed by the QUIMBI visualization.

### Effect of ProViM on QUIMBI visualizations

**Figure 9:**
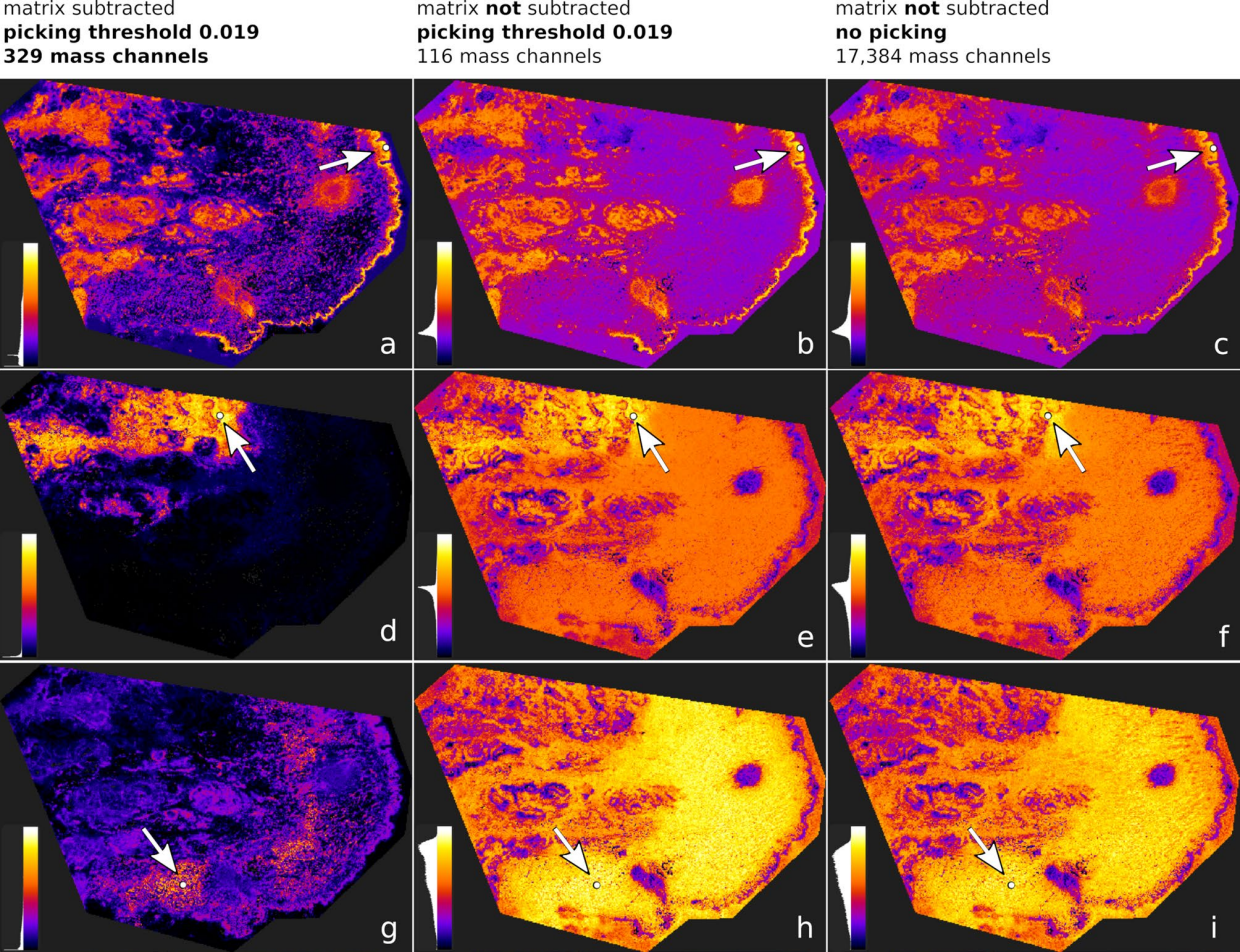
Comparison of QUIMBI visualization with and without matrix subtraction. The column on the left (**a**), (**d**), (**g**) shows the QUIMBI visualization of three different reference pixels (marked with arrows) in the human PXE skin data set, which was fully processed with ProViM. The matrix signal was subtracted and the picking threshold was set to 0.019, resulting in 329 mass channels. The columns in the middle (**b**), (**e**), (**h**) and on the right (**c**), (**f**), (**i**) show the QUIMBI visualizations at the same pixel position of the same data set but without matrix subtraction. The image intensity scales in all images show the similarity of pixels from black (dissimilar) to white (similar). In the middle, mass channels with the same threshold value of 0.019 were picked, resulting in 116 mass channels. For the right column no picking was performed, resulting in 17,384 mass channels in the QUIMBI visualization. The best color contrast between the similar and dissimilar pixels in the QUIMBI visualization is achieved when a matrix subtraction was performed.

Using QUIMBI we also investigated the effect of subtracting the matrix signals with ProViM (see Fig. 9) using the human PXE skin data set $I^\text {t}_\text {s}$ as example. The left column (Fig. 9a,d,g) shows the QUIMBI visualizations of three different reference pixels (marked with arrows) in $I^\text {t}_\text {s}$, which was fully processed with ProViM. The matrix signal was subtracted and the picking threshold was set to 0.019, resulting in 329 mass channels. The middle column (Fig. 9b,e,h) and the right column (Fig. 9c,f,i) show the QUIMBI visualizations at the same pixel position of $I^\text {t}_\text {s}$ without matrix subtraction. In the middle, mass channels with the same threshold value of 0.019 were picked, resulting in 116 mass channels. For the right column no picking was performed, resulting in 17,384 mass channels. For the first and second example of the QUIMBI visualization (Fig. 9a–f) the same distribution patterns are visible, even in the data set versions without matrix subtraction, but with much lower color contrast between the similar and dissimilar pixels than those with matrix subtraction. The third example (Fig. 9g,h,i) shows a weak distribution that is just visible when the matrix is subtracted and remains invisible when the matrix is kept in the data set. For all selected reference pixels, the visualization of the ProViM-processed data set shows the best color contrast between the similar and dissimilar pixels for the resulting distribution, i.e. there are fewer similarity values in the middle part of the histogram and more at the two ends.

## Conclusion

In this work we introduced the two software tools ProViM and QUIMBI and demonstrated how those can be combined into a new method for visual MSI analysis. ProViM allows the transformation of raw data into ready-to-visualize data by performing pre-processing steps and subtracting tissue-unspecific signals from the data. The option of subtracting manually or automatically gives users full control about this sensitive step in data pre-processing. However, as the matrix and artifacts subtraction step is data driven, i.e. based on the projection of the original data, the quality of the results must be checked as there is always a theoretical chance of introducing a bias in the data. In our use cases we did not observe such a problem though.

The web-based visualization tool QUIMBI is interactive, easy to use and requires almost no training. It allows to browse across the spatial dimension of the sample in real time and to examine the co-location similarity distributions for the pixel mass spectra. It scales well along the lateral dimension and is capable of handling large data sets with a high spectral resolution.

Our use cases showed that QUIMBI allows the examination and identification of layers and structures in a tissue section without the experience of a pathologist. Since the interactive exploration is performed on the spatial dimension, i.e. the sample’s morphology, instead of the mass spectrum, interesting distribution patterns become visible which could not be found by examining only single or multiple mass channels. Since the QUIMBI visualization does not use a data reduction step (such as dimension reduction or clustering) apart from the required 8-bit transformation and it has the potential for easy interpretation, it provides a useful new way for the detection of interesting structures and hidden regularities in tissue sections. Such patterns can be difficult (or maybe impossible) to detect for the untrained eye in classically stained tissue sections, such as the calcified layer in PXE skin, presented in this work. A QUIMBI visualization quickly highlights areas of similar molecular composition. In research pathology, this rapid information gain could help to understand the (molecular) nature of (unknown) regions and in clinical pathology the use of QUIMBI could support and simplify the pathologist’s work by accelerating the process of identifying regions of similar molecular co-location.

## Material and methods

### Tissue preparation and staining

All experiments within this study were performed in accordance with relevant guidelines and regulations. The experimental work in this study was performed with dead animals. The mice were killed exclusively for the use of their organs or tissues without any incriminating experiments. Therefore, the animal preparations comply with the German Animal Welfare Law used for scientific purposes, ethical approval for our study was not required. The human skin sample is part of a study approved by the ethics committee of the HDZ NRW, Department of Medicine, Ruhr-University Bochum (registration number 32/2008). All patients gave their written informed consent to participate in the study.

Fresh vibrissae and kidney biopsy tissue samples from male mice and a human PXE skin tissue sample were rapidly frozen in liquid nitrogen, cut into 10 $\upmu \text {m}$ sections in a cryostat (Leica Biosystems) and mounted on indium tin oxide (ITO) coated glass slides with further drying in a desiccator for at least 30 min. Subsequent tissue sections were mounted on a glass slide (SuperFrost) for histological staining. 2,5-dihydroxybenzoic acid was prepared as 30 $\text {mg mL}^{-1}$ in 50% Methanol and 1% trifluoroacetic acid and sprayed onto tissues with a TM-Sprayer (HTX Technologies, LLC) using parameters of 75$^\circ$C, 10 psi, 0.1 $\text {mL min}^{-1}$, 1200 $\text {mm min}^{-1}$ and 8 passes in a crisscross pattern with a 3 mm spacing.

A 2% Alizarin Red S staining solution was prepared in distilled water and pH was adjusted to 4.1–4.3 with 10% ammonium hydroxide. The staining solution was applied onto the vibrissae and human PXE skin tissue sections and incubated at room temperature for 10 min. The staining solution was removed, the slide was first dehydrated in 100% acetone (20 dips) and then in acetone/xylol (1:1n) solution (20 dips). The slide was cleared in xylene and afterwards mounted in a synthetic mounting medium.

HE-staining was applied to the subsequent human PXE skin and mouse kidney tissue sections. Ready-to-use solutions of Mayer’s acidic hemalaun and aqueous 0.5% eosin G solution (Carl Roth) were filtered before usage. Both tissue sections were fixed in 100% methanol (2 min), followed by a washing step (10 dips) with demineralized water ($\text {dH}_2$O). The acidophilic structures of the tissue were stained with hemalaun solution (6 min). A second washing step with $\text {dH}_2$O followed before blueing under running tap water (10 min). Aqueous 0.5% eosin G solution was used after a short washing step with $\text {dH}_2$O to counterstain the basophilic structures (8 s). Further washing and differentiation steps were performed with fresh 100% ethanol (2 × 10 dips). The slides were cleared in xylene and mounted using a synthetic mounting medium.

### Mass spectrometry imaging

One kidney and one human PXE skin tissue section on ITO slides were analyzed by MALDI-ToF-MSI (ultrafleXtrem; Bruker) in positive ion mode, collecting 350 laser shots per pixel and a laser frequency of 1000 Hz. Laser global offset was set to 71% and laser energy was set to 60% for the kidney sample. Detector voltage was set to 1200 V. Spatial resolution was 20 $\upmu \text {m}$ and laser diameter was “medium”. Data was exported to imzML by flexImaging 4.1. The mouse vibrissae and kidney tissue sections on ITO slides were analyzed by MALDI-Orbitrap-MSI (Q Exactive Plus; Thermo Scientific with MALDI/ESI injector; Spectroglyph, LLC) in positive ion mode. The AGC Target was fixed with an injection time of 250 ms. Diode laser current was set to 2.0 Amps and laser repetition rate was 500 Hz. Spatial resolution was 20 $\upmu \text {m}$ and spectral resolution fixed to 70,000 bins. Raw data and position data were aligned and exported to imzML with Image Insight Software (version 12068).

*Data sets:* The data sets $I^\text {O}_\text {v}$, $I^\text {O}_\text {k}$ and $I^\text {t}_\text {k}$ were reduced to the mass range from 100 to 1150 $m/z$. The data set $I^\text {t}_\text {s}$ was reduced to the mass range from 100 to 1400 $m/z$. Details about numerical features of the four used data sets can be found in Table 3 of Supplementary H.

### Data set descriptions

We define a single mass spectrometry imaging data set *I* as follows: One data set recorded for one tissue section is a multivariate image *I* that consists of $h \in \mathbb {N^+}$ rows and $w \in \mathbb {N^+}$ columns of pixels $p_{i,j}$, with column index $i \in \{1, \dots , w\}$ and row index $j \in \{1, \dots , h\}$. The set of all pixels is defined as $p = \{p_{i,j}\}$. We define the set of spectral pixels $p^+ = \{p_{i^+, j^+}\} \ \subseteq \ p$ as all positions where the MALDI instrument conducted a local mass spectrometry measurement.

Each spectral pixel consists of $d \in \mathbb {N^+}$ intensity values, each of which corresponds to one mass to charge ratio value ($m/z$), also called mass channel, which approximates a molecular mass. A data point $p_{i,j,k}$ is the intensity value of the *k*-th mass channel of the pixel $p_{i,j} = \{p_{i,j,1}, \dots , p_{i,j,d}\}$, if $p_{i,j} \in p^+$ and zero, otherwise. The mass channel image of the *k*-th mass channel of the multivariate image *I* is defined as $I_k = \{p_{1,1,k}, \dots , p_{w,h,k}\}$. A peak $\rho _{i,j,v} = \sum _{u=l}^{r} (p_{i,j,u})$ is defined as the sum of intensities of a set of mass channels $\{l, \dots , r\}$, where *v* is defined as the median of $\{l, \dots , r\}$ and $l,r \in \{0, \ldots , d\}$ are the first mass channel left and right of the maximum of the peak with an intensity of 50% of the maximum height of the peak, respectively. In this study we do not pick peaks on spectra of individual pixels and therefore define a peak of the mean spectrum $\mu = \frac{1}{|p^+|}\sum _{i^+} \sum _{j^+} p_{i^+,j^+}$ of all pixels as $\rho _v$ and the set of all peaks as $\rho = \{\rho _{v}\}$.

### ProViM

ProViM is based on very general and cost efficient algorithmic approaches. Mass spectrometry imaging data is usually stored in the vendor independent raw formats .ibd and .imzML^19^. Various processing steps are needed to prepare this raw data for downstream analysis. It is possible to execute the entire processing fully automatically. However, the major strength of ProViM is that manual intervention is possible for the very sensitive steps of matrix and artifact subtraction, as well as for peak picking and deisotoping. ProViM is open source under the GNU GPLv3 license and available at https://github.com/Kawue/provim.

A list of all parameters can be found in Table 1 of Supplementary E. In the ProViM tool provided with this manuscript, parameters and options can be easily set by the user.

#### (a) Spectra alignment and normalization

To align and normalize all spectra, we use the respective functions of another recently published tool called pyBASIS^8^. The alignment procedure uses a kernel-based clustering approach to align identical or similar ion species to a common $m/z$ vector. For normalization, pyBASIS offers an intra-sample normalization and a inter-sample normalization. The intra-sample normalization should always be applied as it reduces the variability of intensity values within a data set, which is a commonly known inherent problem of MSI data. The method transforms each spectrum by a scaling factor, specifically set for the spectrum. The inter-sample normalization should be applied additionally when a set of samples is analyzed within a comparative study. The method transforms each spectrum of the same sample by a sample-specific scaling factor. For both methods, three different approaches can be chosen in pyBASIS to calculate the scaling factors: mean, median and median fold change (default). For further details about pyBASIS we refer the interested reader to the original work^8^.

#### (b) Data format reduction

The results of pyBASIS in step (a) are stored in the HDF5 data format^20^ and all information about the measurement is written to two files. The first one contains the raw data *I*. The second one contains the data set after spectral alignment and normalization ${\widetilde{I}}$, plus additional meta information about the chosen processing methods and applied parameters. Data stored in HDF5 files is organized in a folder-like manner. While the HDF5 format has a superior performance compared to imzML^21^, there are currently no standards for the structural organization of MSI data in the HDF5 format. To allow easier programmatic access via the pandas software package, the data ${\widetilde{I}}$ is transformed into a multi-indexed $|p^+| \times d$ pandas DataFrame^22^ $I'$. That way, $I'$ contains only the minimum necessary data for further downstream analysis. Each row represents a triplet ($i^+$, $j^+$, data set ID) and each column as the $m/z$ value. For comparative analysis the option to set a lower and upper $m/z$ cutoff is provided, which is useful if the data sets to be compared have different $m/z$ ranges. This step also recreates .ibd and .imzML files from $I'$ to establish compatibility with other software packages and tools.

#### (c) Matrix and artifact detection and subtraction

The pixels $p^+$ of an MSI data set and their associated spectra can be classified into three categories: (A) sample signals, (B) matrix signals and (C) artifact signals. A and B can be defined as pixels that have a physical location inside the sample or matrix area. Category C are pixels that are classified as artifacts because their spectra are highly different from all sample and matrix spectra. Typically such artifacts appear as single intensity hotspots in the spatial pixel grid. These hotspots are unlikely to represent molecular distributions, which is the reason why we refer to them as artifacts. To detect and subtract pixels of the categories B and C, we developed a module based on non-linear dimension reduction. The module works in three steps:

The first step calculates the embedding of the *d*-dimensional pixels into a two dimensional subspace. Across many alternatives, t-SNE^23^ and UMAP^14^ showed the topologically best resolved results^16^, i.e. they showed the most distinguishable cluster structures. This is illustrated by an exemplary comparison between PCA, NMF, tSVD and UMAP in Supplementary L. Because of its runtime advantage, we have chosen UMAP as the default method over t-SNE. If the number of $m/z$ channels *d* exceeds a threshold of 1000, the linear dimension reduction technique truncated singular value decomposition (tSVD)^11^ is applied as an additional step before UMAP. In this way, we further reduce the computation time without strongly impairing the quality of the UMAP embedding.

In the second step, the artifacts and matrix signals are detected. This detection can be done automatically (see Supplementary J) or interactively to have full control about this subtraction step (see Supplementary B for details).

If pixels are classified as matrix in the previous step, the third step will calculate their mean spectrum and subtract it from every pixel spectrum in the set $p^+$ of data set $I'$. In the third step, the mean spectrum of all pixel spectra that were classified as matrix is calculated and subtracted from the spectrum of all pixels $p_{i^+,j^+}$ of the data set $I'$. Optionally, the same can be done for those pixels classified as artifacts. To avoid negative values, all spectra are limited to a lower limit of zero. Depending on the downstream analysis, matrix and artifact pixels can have a negative impact on the applied algorithms. Therefore, we included the option to remove matrix and artifacts pixels entirely from the set of spectral pixels $p^+$ of the data set. The matrix and artifacts subtraction (and potential pixel removal) transforms $I'$ into the cleaned data set $I''$ (examples in Supplementary A, B).

If strong artifacts are present, it can be helpful to select and remove the artifacts first and to calculate a new embedding without artifacts in a second run. This may apply, for example, if the artifacts have a very large distance to other pixels in the high dimensional space. In UMAP this usually results in a large Euclidean distance in the embedded space, which in turn can have a negative effect on the global structure of the embedding of sample and matrix pixel, i.e. they may be harder to differentiate.

In this manuscript, the entire artifact and matrix subtraction process was performed interactively and separately for all four data sets.

#### (d) Peak picking and deisotoping

The ability to select a subset of high intensity mass channels, referred to as “peak picking”, is an important tool for the analysis of mass spectrometry data. For this reason, ProViM offers a module for peak picking and also contains an option for the removal of isotope signals (deisotoping). Peak picking significantly reduces spectral dimensionality, eliminates noise and is an effective way to discover potentially interesting signals. Therefore, using a picked data set for downstream analyses leads to faster runtimes and less demand on computational resources, allows a more targeted analysis and computed results are often more informative and relevant.

ProViM’s peak picking function can be applied, either automatically or interactively to have full control about this filtering step. An example of the interactive peak picking tool can be found in Figures 3 and 4 of Supplementary C, which also includes additional information about the tool functions. The peaks are determined by a local maximum detection procedure on the mean spectrum. The filtering is realized by defining a minimum peak height $z_t$. Each peak must be above $z_t$ to be selected. Using the interactive version of this module, the mean spectrum, the threshold $z_t$ and the selected peaks are visualized and $z_t$ can be manually adjusted. The module also offers a fixed value based upper limit winsorizing that transforms all data points of the mean mass spectrum $\mu$ to $\mu '_{k} = \min (\omega , \mu _{k})$, where $\omega$ chosen by $z_{w} \in {\mathbb {N}}$ is the $z_{w}$ highest value in $\mu$. This way, high intensity peaks can be clipped to improve the visual representation of the mean spectrum for picking purposes.

As stated above, this module detects peaks using a combination of local maxima and minimum intensity without including other peak characterizing features such as shape and area. This has the advantage to be highly efficient, which is especially important for the interactive variant, as it requires a fast visual response. If necessary, the peak detection could also be extended with more complex methods^24–27^.

For this study, the interactive peak picking was first applied to data set $I^\text {O}_\text {v}$. An intensity threshold of 0.005 was chosen manually to eliminate most of the noise in the data set (see Figure 4 of Supplementary C). Thereafter, this threshold was used for automatic peak picking in the data sets $I^\text {O}_\text {k}$ and $I^\text {t}_\text {k}$. For the data set $I^\text {t}_\text {s}$ an intensity threshold of 0.019 was chosen. We have refrained from the application of deisotoping, because the inclusion of isotope patterns had a positive influence on the visualization quality of QUIMBI.

### ProViM configuration

All four data sets were processed with our ProViM tool. Details about the used parameters and performance measurements can be found in Supplementary E and Supplementary I, respectively.

### QUIMBI

QUIMBI is an interactive visual MSI exploration and analysis tool. It provides information about the similarities of molecular compositions between spectra, which are visualized within the morphological sample space. QUIMBI offers three different visualization modes: (1) *Similarity*, (2) *Browsing* and (3) *Grouping*. Here we provide an overview about the user interface and explain the three visualization modes. Technical details about the implementation can be found in Supplementary K.

#### User interface

The user interface of QUIMBI consists of two displays which are functionally interlinked: the *main panel* (Fig. 4a) and the *spectrum viewer* (Fig. 4b). The main panel displays the main visualization in which each pixel $p_{i,j}$ is represented by an intensity value $c_{i,j}$. Each visualization mode computes an intensity value $c'_{i,j}$ which is normalized to $c''_{i,j}$ (see Eq. 2) and subsequently transformed using a color scale ${\mathcal {C}}$^28^ to produce the final value $c_{i,j}$ for the visualization (see Eq. 1). Currently, QUIMBI uses the “Fire” color scale. Appropriate alternatives that could be considered are, for example, the popular “Magma” or “Viridis” color scales^29^. Computation of the intensity values is based on the byte representation $p'_{i,j}$ of a pixel $p_{i,j}$ (see Supplementary K). For each visualization mode, the intensity values $c'_{i,j}$ are computed as follows:

- *Similarity mode:* The user can hover the mouse over the visualization image or select a single pixel with a click to set the position (*q*, *r*) of the reference pixel $p_{q,r}$. The intensity value $c'_{i,j}$ for each pixel $p_{i,j}$ is computed as the inverse angle between the pixel $p_{i,j}$ and the reference pixel $p_{q,r}$ (see Eq. 3). In this mode, the spectrum viewer shows the spectrum of the selected reference pixel. If no reference pixel is selected, the mean spectrum of the data set is displayed. The inverse angular distance is used for the following reasons reasons. First, it is fast to compute which is an essential feature for an interactive visualization tool. Second, in this work we are less interested in the magnitude of signals but in the peak profile of the spectra, i.e. the orientation of the *n*-dimensional vector described by the peak intensities. An angular metric like the inverse angular distance is suited to distinguish small changes in the peak profile, i.e. small angular differences between two mass spectra. It is also strongly related to the cosine similarity which is commonly used in the MSI community^30–33^ and has shown to be a robust method for mass spectra similarity evaluation in various different works^34–36^.
  Of course it must be noted, that in principle other distance functions could be applied to compute pairwise (dis-)similarities between spectra in the QUIMBI visualization approach. Such functions could have benefits in highlighting other particular aspects of spectral similarity compared to the inverse angular distance applied here. The choice of the inverse angular distance function is driven by the reasons listed above and not by any claim, that this function outperforms other functions for spectral analysis in general.
- *Browsing mode:* The user can hover over the mass spectrum in the spectrum viewer to select a mass channel *k*. In this mode, the intensity value $c'_{i,j}$ is set to equal the data point $p'_{i,j,k}$ (see Eq. 4).
- *Grouping mode:* The user can select one or more ranges of mass channels through a cursor drag interaction in the spectrum viewer. In this mode, the intensity value $c'_{i,j}$ is computed as the mean of all data points of the set of selected mass channels $M \subseteq \{1, \dots , d\}$ at the same location (see Eq. 5).

It is important to note that the displayed mass spectra in the spectrum viewer do not represent the original intensity pattern of the mass channels. This is due to the 8-bit transformation of the data. We chose to display the transformed mass spectra used to compute the QUIMBI visualization instead of the original mass spectra because we put our focus on the visualization. Therefore, the spectrum viewer always shows the exact values that are used to compute the visualization. Although the mass spectrum of a reference pixel does not reflect the original intensities, it is still possible to identify the mass channels responsible for a particular distribution pattern through a QUIMBI visualization and go back to the original data set with this information to obtain the true intensity values.

1 $$\begin{aligned} c_{i,j}= & {} {\mathcal {C}}(c''_{i,j}) \end{aligned}$$

2 $$\begin{aligned} c''_{i,j}= & {} 255 \cdot \frac{c'_{i,j} - \min _{i,j} c'_{i,j}}{\max _{i,j} c'_{i,j} - \min _{i,j} c'_{i,j}} \end{aligned}$$

3 $$\begin{aligned} c'_{i,j}= & {} \left [ 255 \cdot \left( 1 - \frac{2}{\pi } \cdot \arccos {\frac{p'_{i,j} * p'_{q,r}}{|p'_{i,j}| \cdot |p'_{q,r}|}} \right) \right ], \text {where} * \text {is the dot product}, [\cdot ] \text { is rounding} \end{aligned}$$

4 $$\begin{aligned} c'_{i,j}= & {} p'_{i,j,k} \end{aligned}$$

5 $$\begin{aligned} c'_{i,j}= & {} \Big [ 255 \cdot \frac{1}{|M|} \cdot \sum _{k \in M}{p'_{i,j,k}} \Big ] \end{aligned}$$

By visualizing the peak pattern similarity (Eq. 3) in the lateral space, *Similarity Mode* enables a perception of the relations between peak pattern similarity and morphology^17^. *Browsing Mode* allows the exploration of single mass channel images, to identify mass channels with interesting lateral patterns. Finally, *Grouping Mode* allows to explore the distribution of combined mass channels through the range selection. This supports the identification of mass channels with similar lateral distributions. Another aim could be to find a combination of mass channels that may cover a specific area of interest.

While browsing the spectrum, an associate annotation appears which shows the exact $m/z$ value and intensity of the measured point closest to the cursor position. We also enhanced the color scale legend by a histogram plot (Fig. 4c), which shows the current similarity/intensity value distribution.

QUIMBI is implemented in WebGL to run in real time in a web browser. Consequently, it works independently of the operation system and requires only a browser. QUIMBI is open source under the GNU GPLv3 license and available at https://github.com/BiodataMiningGroup/quimbi. Details about the implementation can be found in Supplementary K.

## Supplementary Information

Supplementary Information.

## Data Availability

QUIMBI is available at https://github.com/BiodataMiningGroup/quimbi under the GNU GPLv3 license. ProViM is available at https://github.com/Kawue/provim under the GNU GPLv3 license.

## References

[1] Herold J, Loyek C, Nattkemper TW (2011). Multivariate image mining. Wiley Interdiscip. Rev. Data Min. Knowl. Discov..

[2] Fonville JM (2012). Robust data processing and normalization strategy for MALDI mass spectrometric imaging. Anal. Chem...

[3] Race AM (2016). Spectralanalysis: Software for the masses. Anal. Chem..

[4] Bokhart MT, Nazari M, Garrard KP, Muddiman DC (2018). Msireader v1. 0: Evolving open-source mass spectrometry imaging software for targeted and untargeted analyses. J. Am. Soc. Mass Spectrom..

[5] Klinkert I, Chughtai K, Ellis SR, Heeren RM (2014). Methods for full resolution data exploration and visualization for large 2d and 3d mass spectrometry imaging datasets. Int. J. Mass Spectrom..

[6] Ràfols P (2017). rMSI: An R package for MS imaging data handling and visualization. Bioinformatics.

[7] Bemis KD (2015). Cardinal: An r package for statistical analysis of mass spectrometry-based imaging experiments. Bioinformatics.

[8] Veselkov K (2018). Basis: High-performance bioinformatics platform for processing of large-scale mass spectrometry imaging data in chemically augmented histology. Sci. Rep..

[9] Neldner KH (1988). Pseudoxanthoma elasticum. Int. J. Dermatol..

[10] Ladewig M (2006). Pseudoxanthoma elasticum. Der Ophthalmologe.

[11] Halko N, Martinsson P-G, Tropp JA (2011). Finding structure with randomness: Probabilistic algorithms for constructing approximate matrix decompositions. SIAM Rev..

[12] Pedregosa F (2011). Scikit-learn: Machine learning in Python. J. Mach. Learn. Res..

[13] scikit-learn. https://scikit-learn.org/stable/index.html. [Version: 0.20.2].

[14] McInnes, L., Healy, J. & Melville, J. Umap: Uniform manifold approximation and projection for dimension reduction. *arXiv preprint* arXiv:1802.03426 (2018).

[15] Umap: Uniform manifold approximation and projection for dimension reduction. https://umap-learn.readthedocs.io/en/latest/. [Version: 0.3.9 ].

[16] Smets T (2019). Evaluation of distance metrics and spatial autocorrelation in uniform manifold approximation and projection applied to mass spectrometry imaging data. Anal. Chem..

[17] McCombie G, Staab D, Stoeckli M, Knochenmuss R (2005). Spatial and spectral correlations in MALDI mass spectrometry images by clustering and multivariate analysis. Anal. Chem..

[18] Gorzolka K, Kölling J, Nattkemper TW, Niehaus K (2016). Spatio-temporal metabolite profiling of the barley germination process by MALDI MS imaging. PLoS ONE.

[19] Schramm T (2012). imzML—a common data format for the flexible exchange and processing of mass spectrometry imaging data. J. Proteomics.

[20] The HDF Group. Hierarchical Data Format, version 5 (1997-NNNN). http://www.hdfgroup.org/HDF5/.

[21] Wilhelm M, Kirchner M, Steen JA, Steen H (2012). mz5: Space-and time-efficient storage of mass spectrometry data sets. Mol. Cell. Proteomics.

[22] McKinney, W. *et al.* Data structures for statistical computing in python. In *Proceedings of the 9th Python in Science Conference*, Vol. 445, 51–56 (Austin, 2010).

[23] Maaten Lv. d., Hinton G (2008). Visualizing data using t-SNE. J. Mach. Learn. Res..

[24] Yang C, He Z, Yu W (2009). Comparison of public peak detection algorithms for MALDI mass spectrometry data analysis. BMC Bioinform..

[25] Wehofsky M, Hoffmann R, Hubert M, Spengler B (2001). Isotopic deconvolution of matrix-assisted laser desorption/ionization mass spectra for substance-class specific analysis of complex samples. Eur. J. Mass Spectrom..

[26] Slawski M (2012). Isotope pattern deconvolution for peptide mass spectrometry by non-negative least squares/least absolute deviation template matching. BMC Bioinform..

[27] Picaud V (2018). Linear MALDI-ToF simultaneous spectrum deconvolution and baseline removal. BMC Bioinform..

[28] Wong, B. Points of View: Color coding (2010). 10.1038/nmeth0810-573 20704014

[29] Matplotlib colormaps. https://bids.github.io/colormap/. [Online; accessed 12-September-2019].

[30] Dexter A (2017). Two-phase and graph-based clustering methods for accurate and efficient segmentation of large mass spectrometry images. Anal. Chem..

[31] Dexter A, Race AM, Styles IB, Bunch J (2016). Testing for multivariate normality in mass spectrometry imaging data: A robust statistical approach for clustering evaluation and the generation of synthetic mass spectrometry imaging data sets. Anal. Chem..

[32] Winderbaum LJ (2015). Feature extraction for proteomics imaging mass spectrometry data. Ann. Appl. Stat..

[33] Zhvansky E (2019). Metrics for evaluating the stability and reproducibility of mass spectra. Sci. Rep..

[34] Stein SE, Scott DR (1994). Optimization and testing of mass spectral library search algorithms for compound identification. J. Am. Soc. Mass Spectrom..

[35] Wan KX, Vidavsky I, Gross ML (2002). Comparing similar spectra: From similarity index to spectral contrast angle. J. Am. Soc. Mass Spectrom..

[36] Kim, S. & Zhang, X. Comparative analysis of mass spectral similarity measures on peak alignment for comprehensive two-dimensional gas chromatography mass spectrometry. *Comput. Math. Methods Med.* **2013**, (2013). 10.1155/2013/509761 PMC3787630 24151524

