# Supplementary material for: Fast visual exploration of mass spectrometry images with interactive dynamic spectral similarity pseudocoloring
Source: Sci Rep. 2021 Feb 25;11:4606. doi: 10.1038/s41598-021-84049-4 (PMC7907387; doi:10.1038/s41598-021-84049-4)
Supplement: Supplementary file 1 — Supplementary Information. [file 41598_2021_84049_MOESM1_ESM.pdf]

Fast Visual Exploration of Mass Spectrometry Images with Interactive Dynamic Spectral Similarity Pseudocoloring

Karsten Wüllems[1,2,3,+,*], Annika Zurowietz[3,4,+,*], Martin Zurowietz[2], Roland Schneider[3,4], Hanna Bednarz[3,4], Karsten Niehaus[3,4], and Tim W. Nattkemper[2,3]

[1]International Research Training Group ”Computational Methods for the Analysis of the Diversity and Dynamics of Genomes”, Bielefeld University , Bielefeld , 33615 , Germany
[2]Biodata Mining Group, Faculty of Technology , Bielefeld University , Bielefeld , 33615 , Germany
[3]Center for Biotechnology (CeBiTec), Bielefeld University , Bielefeld , 33615 , Germany
[4]Proteome and Metabolome Research, Faculty of Biology, Bielefeld University , Bielefeld , 33615 , Germany
[*]wuellems@cebitec.uni-bielefeld.de, annika@cebitec.uni-bielefeld.de
[+]these authors contributed equally to this work

Supplementary

A Interactive Matrix Subtraction

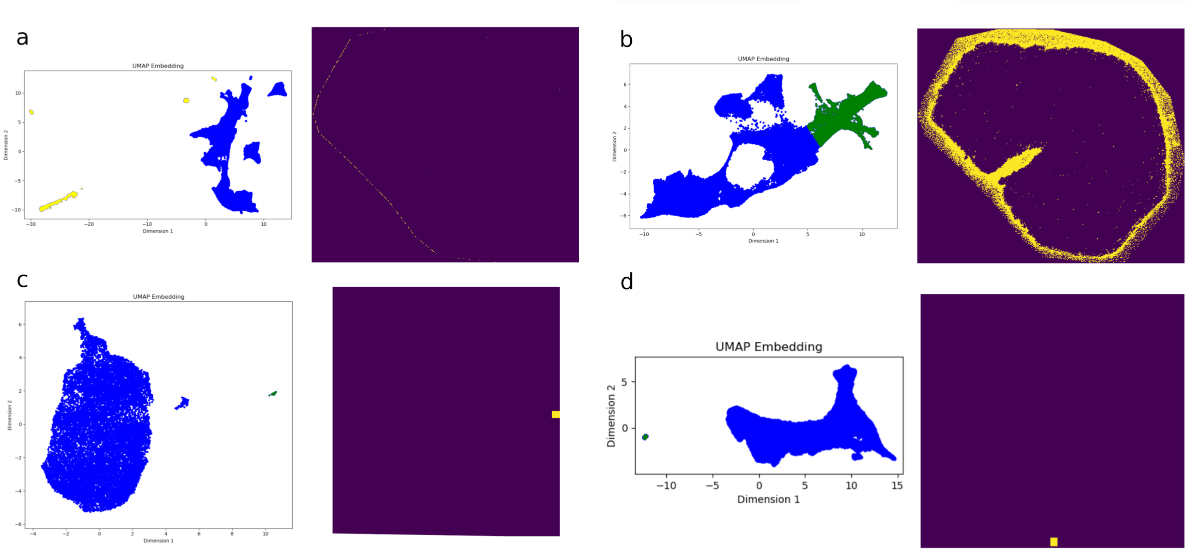

Figure 1. Interactive artifact and matrix subtraction. UMAP embedding (scatter plots on the left) with sample pixels marked in blue, artifact pixels are marked in yellow and matrix pixels in green. The corresponding pixels are shown on the right, respectively. (a) I_k^O artifact subtraction (b) I_k^O matrix subtraction (c) I_v^O matrix subtraction (d) I_k^t matrix subtraction. The shown visualization is part of ProViMs interactive artifact and matrix subtraction module. The dimension reduction was computed by a successive combination of tSVD[1–3] and UMAP[4,5] as described in Material and Methods.

B Introduction to the Interactive Matrix Subtraction Tool

When the interactive matrix subtraction tool is started it shows the selected embedding in blue on the left and an empty image on the right (see Figure 2 for an example). By using the respective buttons and a lasso selector in the embedding, the user

can select regions of interest (RoI) represented in orange, matrix pixels represented in green, and artifact pixels represented in yellow. After the lasso has been used, the selected pixels are shown as binary image at the right. Currently, only the last selection is visualized. Therefore, if selections of multiple categories (RoI, matrix, artifacts) are done, only the pixels of the last selection will be shown. The *Reset* button will reset every selection. By using the *Run* button, the remaining workflow will be completed as described in the section **Matrix and artifact detection and subtraction** in the main manuscript. For further downstream analysis of potential interesting regions independent of ProViM, the user can select a RoI, type a label in the *Type Name* field and use the *Export* button. This will export a .npy numpy array[6] which contains the indices of the selected pixels. Those indices can be used to filter either the embedding or the pandas DataFrame of the used data set.

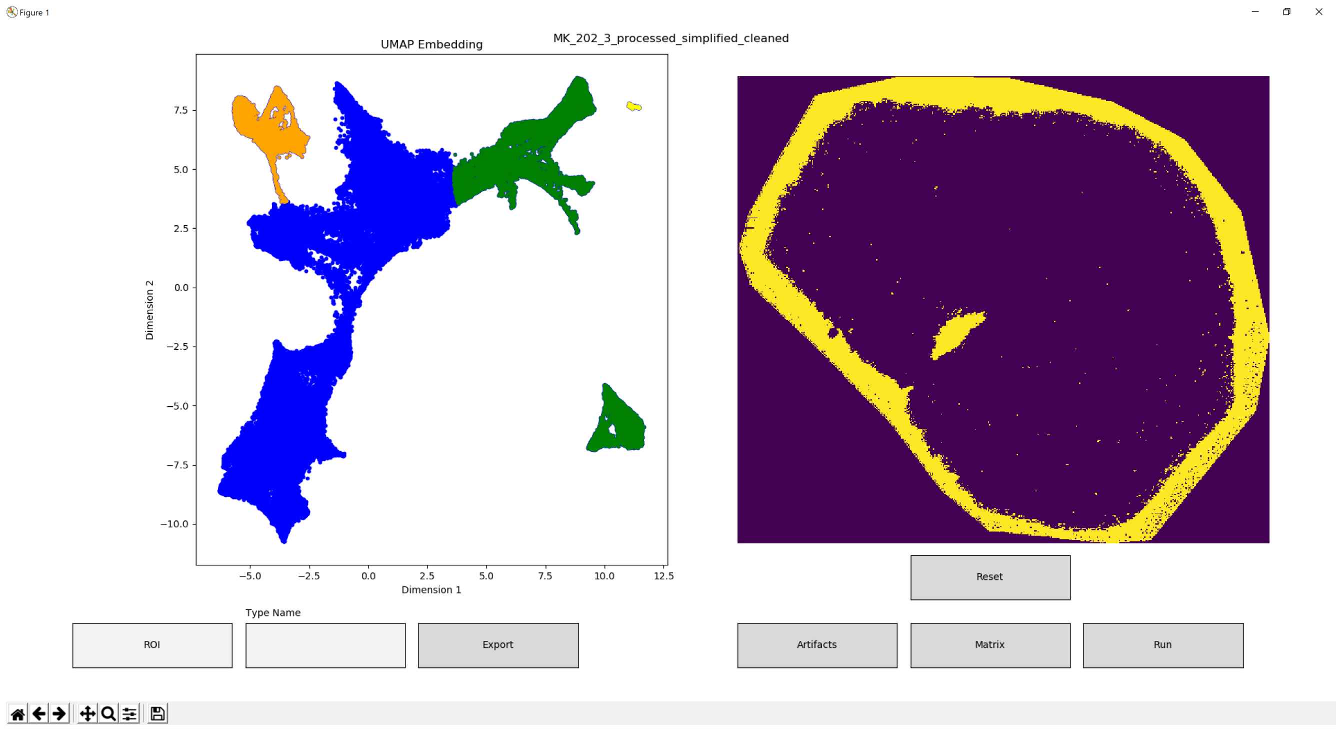

Figure 2. Interactive matrix and artifact detection and subtraction tool. The embedding of all pixels is shown on the left side, with a default color of blue. The binary image of the current pixel selection is shown on the right. In this example, the image corresponds to the selection of matrix pixels. The region of interest selection is represented in orange, the artifact selection in yellow and the matrix selection in green. The shown visualization is part of ProViMs interactive artifact and matrix subtraction module. The dimension reduction was computed by a successive combination of tSVD[1–3] and UMAP[4,5] as described in Material and Methods.

C Introduction to the Interactive Peak Picking Tool

When the interactive peak picking tool is started it shows only the mean spectrum of all pixels (blue) (see Figure 3 for an example). By moving the mouse across the screen, a helper line (green) with a number next to it indicates the currently selected threshold value. A right click activates the peak detection algorithm. After completion, the computed peaks are shown as circles (red) and the number of computed peaks are displayed on the lower left. The default value for deisotoping is 1.2, but can be changed by the user. The *Deiso* button starts the deisotoping algorithm. Peaks that remain after deisotoping are shown as smaller circles (orange) and their quantity is also shown at the lower left. The *Run* button continues the remaining peak picking workflow with the selected threshold.

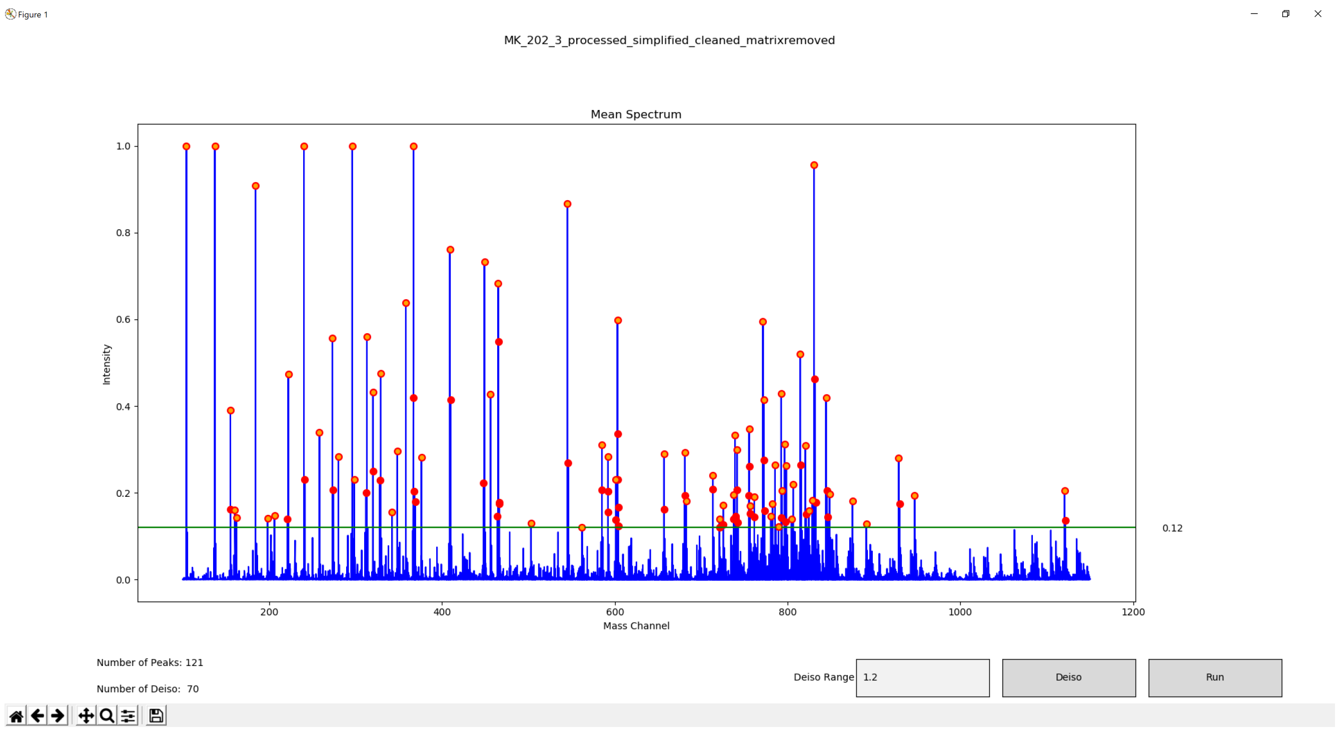

Figure 3. Interactive peak picking tool after threshold selection of 0.12 and deisotoping. The threshold is indicated by the number at the far right and the green helper line. The mean spectrum is shown in blue, peaks as red circles (121 in total, see lower left), peaks that remain after deisotoping as orange circle (70 in total, see lower left). The selected deisotope range is 1.2 .

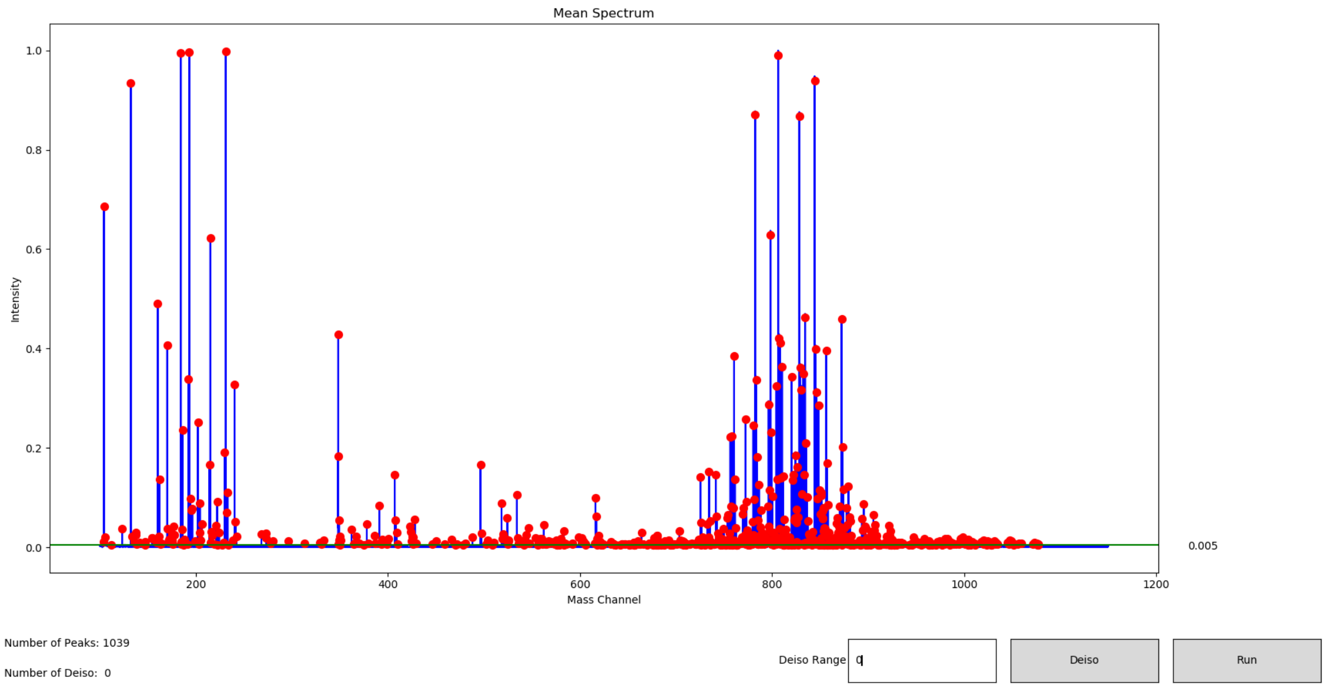

Figure 4. Screenshot of interactive peak picking on data set I_v^O from the picking tool: the average mass spectrum after matrix subtraction is shown in blue. The peak picking threshold z_t was manually set to 0.005 (green line) and all detected peaks (1039) are marked in red. Deisotoping range was set to 0 (off).

D Deisotoping

The deisotoping procedure uses a minimum and maximum peak distance to compute potential isotope sets. These sets are checked for two kinds of patterns. The first one is characterized by a series of peaks that continuously decrease from a maximum to a minimum (descent pattern). The second is characterized by a series of peaks that increase up to a maximum, followed by a decrease (hill pattern). If such patterns are found, either the peak with maximal intensity (default) or the first one of the series is picked and the others are discarded. The algorithm to find these patterns is a two step procedure: 1. Consecutive peaks within a specified distance are computed and collected in a isotope set. 2. Each isotope set is checked whether more than one local maximum is present. If the check is positive, these sets are broken recursively at the local minimum that lies between the first two local maxima. This happens until each set contains only a single local maximum. Although this method is a rough approximation to find isotopes, it has the advantage to be efficient and no prior knowledge is required.

E Table of Parameters

Table 1. Explanation of all customizable parameters and their values used in this study.

Script	Arguments	Used	Meaning
`workflow_pybasis.py`	z_{f1}	local dir	Folder with one or multiple .imzML and .ibd files
	z_{s1}	basis.h5	filename of raw and processed hdf5 files
	z_{in}	False	activation of inter sample normalization
	z_{bin}	0.001	bin size for spectral binning (in Dalton)
	z_{shift}	0.1	maximum allowed shift for the alignment procedure (in Dalton)
`fast_convert_hdf.py`	z_{f2}	local file	Path to processed file of the last step
	z_{s2}	local folder	Path to folder to save results
	z_{l1}	100	Lower m/z channel limit
	z_{l2}	1150	Upper m/z channel limit
`matrix_preprocessing.py`	z_{f3}	local file	Path to processed file of the last step
	z_{s3}	local folder	Path to folder to save results
`interactive_matrix_detection.py`	z_{f4}	local file	Path to processed file of the last step
	z_{s4}	local folder	Path to folder to save results
	z_e	local file	Path to embedding created in the last step
`automated_matrix_detection.py`	z_{f4}	local file	Path to processed file of the last step
	z_{s4}	local folder	Path to folder to save results
	z_{nk}	15	Number of k-nearest neighbors
	z_{nr}	10	Distance radius for k-nearest neighbors
`matrix_postprocessing.py`	z_{f5}	local file	Path to processed file of the last step
	z_{s5}	local folder	Path to folder to save results
	z_a	True	Boolean if mean artifacts spectrum should be removed from the sample spectra, requires artifact detection with the interactive matrix detection tool
	z_{dm}	False	Boolean if artifact pixels spectrum should be removed from the sample, requires artifact detection with the interactive matrix detection tool
	z_{da}	False	Boolean if matrix pixels spectrum should be removed from the sample, requires a detected matrix
`workflow_peakpicking.py`	z_{f6}	local file	Path to processed file of the last step
	z_{s4}	local folder	Path to folder to save results
	z_w	5	Upper winsorizing cutoff.
	z_{pm}	False	Boolean if picking should be done on every data set separately or on a merged version
	z_b	none	Transformation method. Expects ”sqrt” or ”log” or ”none”
	z_{ia}	True/False	Boolean if interactive picking should be used. If z_{iso} and z_t are given they will be ignored
	z_{iso}	0	Isotope range. A float is expected and will be interpreted in dalton
	z_t	0.005	Threshold or a list of thresholds for picking

F Use case 1

Table 2. List of ten masses with the highest signal in the QUIMBI visualizations shown in Figure 5 of the main manuscript.

Ranking	Figure 5(a) I_k^O [m/z]	Figure 5(b) I_k^t [m/z]	Figure 5(c) I_k^O [m/z]	Figure 5(d) I_k^t [m/z]
1	296.07	296.11	830.54	792.66
2	258.11	258.14	771.47	830.64
3	366.96	180.12	792.59	793.67
4	848.56	104.13	831.55	814.66
5	280.10	782.63	814.57	831.64
6	657.00	193.02	928.61	794.67
7	789.48	810.65	815.57	832.65
8	806.51	804.61	793.59	104.13
9	820.52	832.65	772.47	781.86
10	798.54	616.22	755.50	818.68

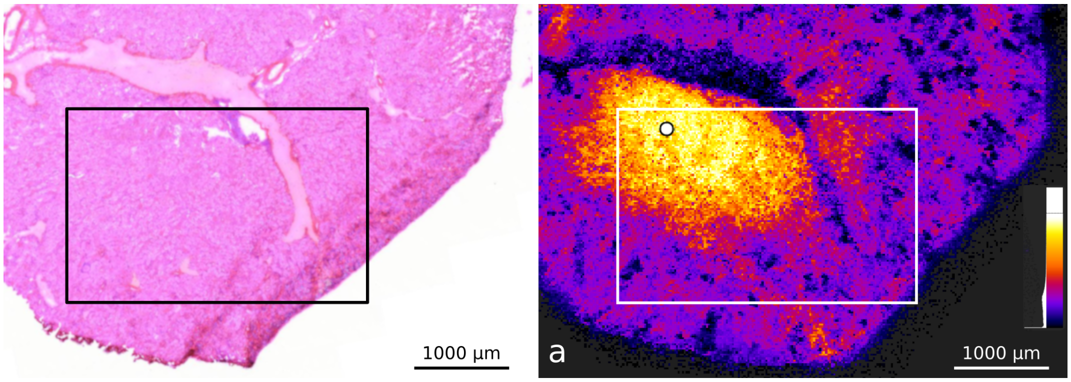

Figure 5. Corresponding HE staining to QUIMBI visualization in Figure 5 of the main manuscript. HE staining was performed on the consecutive section of the kidney sample.

G Use case 2

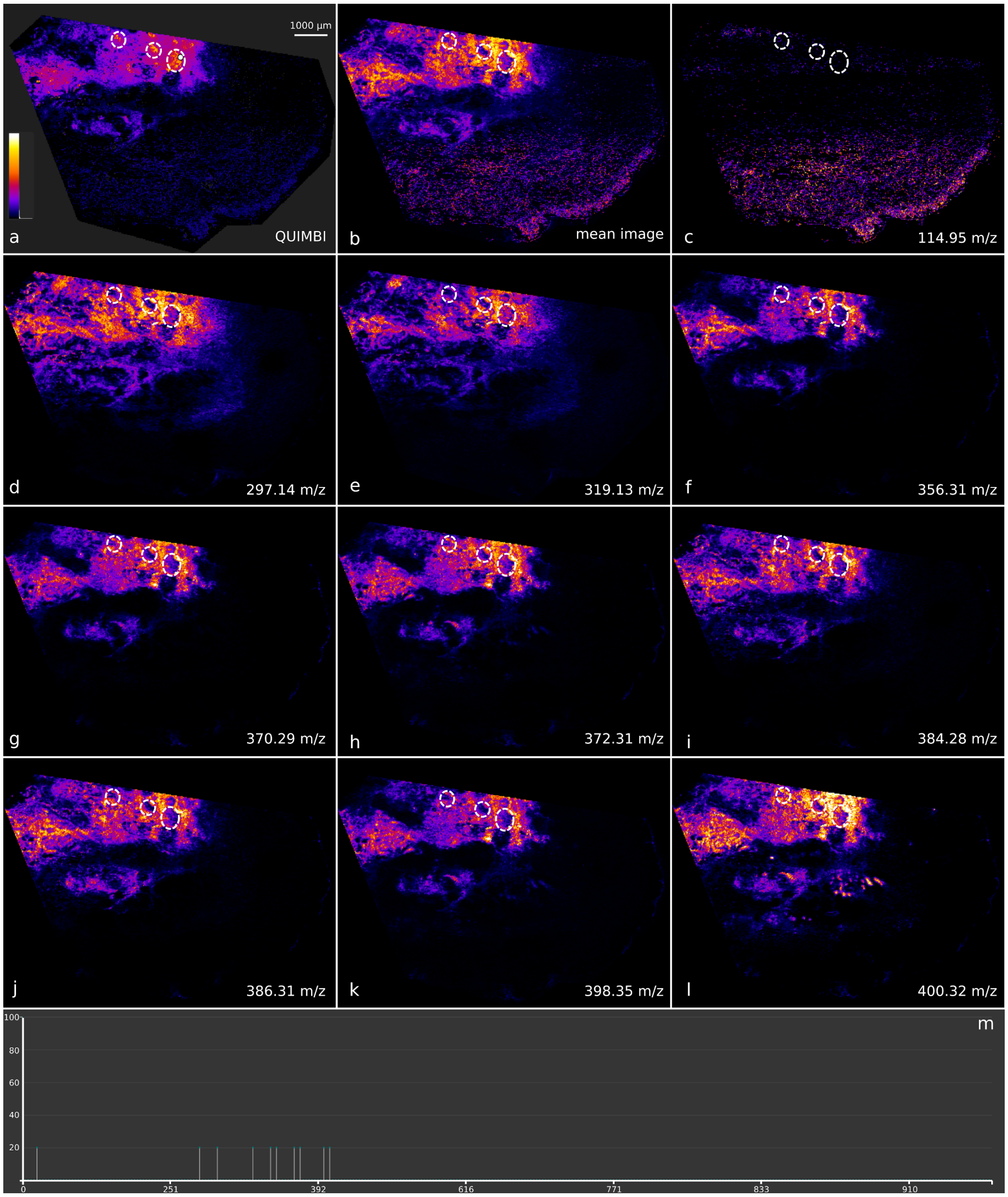

Figure 6. QUIMBI visualization (a) of human PXE skin showing a distinct distribution which is based on weak signals. (b) shows the mean image of the single mass channel images (c)-(l). (m) shows the corresponding mass spectrum of the selected reference pixel in (a). The marked regions (dashed white lines) show a molecular distribution which is visible in the QUIMBI visualization (a) but not in the single mass channel images (c) to (l) or in the mean image (b).

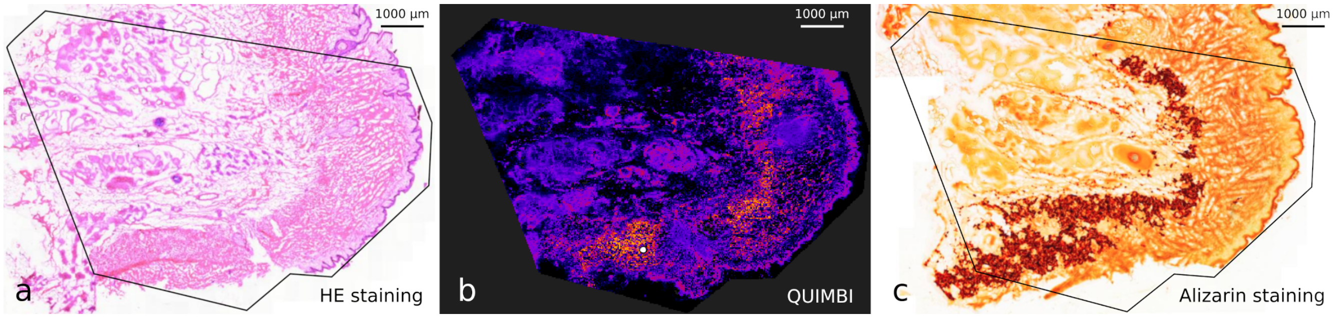

Figure 7. HE staining (a), QUIMBI visualization (b) and Alizarin staining (c) of the human PXE skin tissue sample. The Alizarin staining shows a calicfication area in deep red that corresponds with the highlighted region in the QUIMBI visualization.

H Data Set Features

Table 3. Data set features. The height h and the width w as well as the number of spectral pixels $|p^+|$ and the number of mass channels d are listed.

Data set	h	w	$\|p^+\|$	d
I_v^O	257	181	26571	78249
I_k^t	230	379	24970	39696
I_k^O	483	424	154290	39696
I_s^t	473	385	75210	17382

I ProViM Performance and Statistics

ProViM was executed on a machine with 500 GB memory and 42 CPUs, using the parameters described in the section **ProViM Configuration**. Table 4 of Supplementary I shows the total run time without the interactive parts of artifact and matrix subtraction, peak picking and deisotoping, the maximum CPU usage, and the number of peaks picked before and after matrix subtraction. The average run time was 0.14 $\text{s} \cdot \text{pixel}^{-1}$. The number of peaks after matrix subtraction increased by a factor of 1.2 to 2.2 compared to the number of peaks before matrix subtraction. Depending on the data set, executing ProViM has a high memory consumption (e.g. 22 GB for data set I_s^t and 181 GB for data set I_k^O). Multiple CPUs are also advantageous to speed up the process with parallelization. Accordingly, it is not advisable to run the pipeline on a standard desktop computer.

Table 4. Pipeline statistics for each data set. The number of spectral pixels $|p^+|$, the total run time of ProViM, the maximal CPU usage (100% per core), the maximal memory usage and the number of peaks before $|\rho^m|$ and after the matrix $|\rho^c|$ subtraction with a peak picking threshold $z_t = 0.005$ are shown.

Data set	$\|p^+\|$	run time [min]	peak memory usage [GB]	max CPU usage [%]	$\|\rho^m\|$ $z_t = 0.005$	$\|\rho^c\|$ $z_t = 0.005$
I_v^O	26571	75	65	493	921	1425
I_k^t	24970	44	31	1581	635	749
I_k^O	154290	380	181	1480	1189	1912
I_s^t	17384	37	22	1137	310	680

J Automatic Matrix and Artifact Detection and Subtraction Procedure

In the automatic detection, the embedding is squared and multiplied by its original signs to increase the distance between the embedded pixels. With higher distances between embedded pixels, the parameters can be chosen more generously. Then, a k-Nearest-Neighbors (kNN) graph and a radius-Neighbors (rNN) graph are constructed and connected components are calculated (CC_{kNN}, CC_{rNN}). This approach is similar to the one proposed in[7] for sequencing contamination detection. For the

purpose of matrix classification, the number of connected components $|CC|$ as well as their compositions are compared. To detect the final matrix pixels the following procedure is applied:

1. If $|CC_{kNN}| < 2 \wedge |CC_{rNN}| < 2$: no data is classified as matrix.
2. If $|CC_{kNN}| = 2 \wedge |CC_{rNN}| = 2$: if the compositions of the smaller components are different, return their joint set of pixels as matrix pixels.
3. If $|CC_{kNN}| = 2 \oplus |CC_{rNN}| = 2$: select the respective method and return the pixels of the the smaller component as matrix.
4. If $|CC_{kNN}| > 2 \wedge |CC_{rNN}| > 2$: select the method with fewer components and return the pixels of every component except the biggest one as matrix pixels.
5. If $2 < |CC_{kNN}| = CC_{rNN}$: calculate the joint set of pixels for all components from both methods except their biggest one and return it as matrix.

This automatic classification is not capable to distinguish between artifact pixels and matrix pixels. If matrix and artifacts are present, the algorithm will return the combination of both as matrix. Similar to the interactive matrix and artifact detection and subtraction procedure, new data sets will be created. One containing only the detected matrix pixels (combination of matrix and artifacts) and the other one containing the remaining pixels. This allows access to the detection result for a manual in-depth analysis if required.

K Efficient Implementation of QUIMBI

QUIMBI's user interface is implemented in the JavaScript programming language in combination with the Vue.js framework. It uses the graphics processing unit (GPU) for hardware accelerated computation of the interactive visualizations in real time. In a web-based JavaScript application, the GPU is exposed through the WebGL application programming interface (API). WebGL is intended to be used to display two- or three-dimensional graphics in a web browser. In order to process much higher dimensional data, QUIMBI uses the WebGL API in a special way, which is outlined in the following. A typical graphics rendering pipeline with WebGL consists of four steps:

Step 1: GPU memory allocation and initialization of the WebGL *shader programs* which contain the logic that should be executed in the GPU. The GPU memory is typically used to store *textures*, images that are projected to two- or three-dimensional surfaces when the rendering pipeline is executed. The total size of the available texture memory varies depending on the model of the GPU. However, the texture memory layout is always a two-dimensional array of 4 byte values, one each for the red, green, blue and alpha color channels of an image. To fit a multivariate mass spectrometry data set $I = \{I_1, \dots, I_d\}$, which consists of d mass channel images, into the texture memory, the data points $p_{i,j,k}$ are first transformed to bytes $p'_{i,j,k} \in \{x \in \mathbb{N} | 0 \leq x \leq 255\}$ (see Equation 1). The result is a normalized data set $I' = \{I'_1, \dots, I'_d\}$. The normalized data set I' is split into *tiles* $I'_{l-r} = \{I'_l, \dots, I'_r\}$, where each tile consists of four consecutive mass channels. Each tile can be treated like a regular image, only that the four color channels of the regular image now correspond to four mass channels of the data set. All tiles of a data set can be loaded into the texture memory of the GPU and are available to be accessed by the WebGL *shader programs*.

$$p'_{i,j,k} = \left[255 \cdot \frac{p_{i,j,k}}{\max_{i,j,k}(p_{i,j,k})}\right], \text{where } [\cdot] \text{ denotes the rounding operation} \quad (1)$$

Step 2: A *vertex shader program* computes the scene of two- or three-dimensional shapes. In the case of QUIMBI, this scene consists of only a single two-dimensional rectangle on which the image of the data visualization should be projected for display.

Step 3: A *fragment shader program* computes the color of each pixel of the scene that should be rendered. The *fragment shader program* is executed for each pixel in parallel on the GPU, which makes the computation very fast. The color of a pixel is typically based on a single interpolated color from a texture, as well as computed shadows or reflections in a three-dimensional scene. However, in order to compute the color of a pixel for the data visualization in QUIMBI, the *fragment shader program* needs to access the colors from the appropriate positions of all the tiles I'_{l-r} of the data set, that are stored in texture memory. To compute the color $c_{i,j}$ of a pixel in the data visualization, different *fragment shader programs* are executed, depending on the current visualization mode. Each *fragment shader program* first computes an similarity/intensity value $c'_{i,j}$ which is then transformed to the final color $c_{i,j}$ of the pixel using $\mathscr{C}$ (see Equation 1 of the main manuscript).

Step 4: In this final step of the WebGL rendering pipeline, the image produced by the *fragment shader program* is returned from GPU memory and drawn in the user interface. For the interactive real time visualization, the complete WebGL rendering

pipeline is executed in a loop. The data visualization is updated whenever the mouse position changes (*Similarity Mode*) or the selected mass channels change (*Browsing Mode* and *Grouping Mode*).

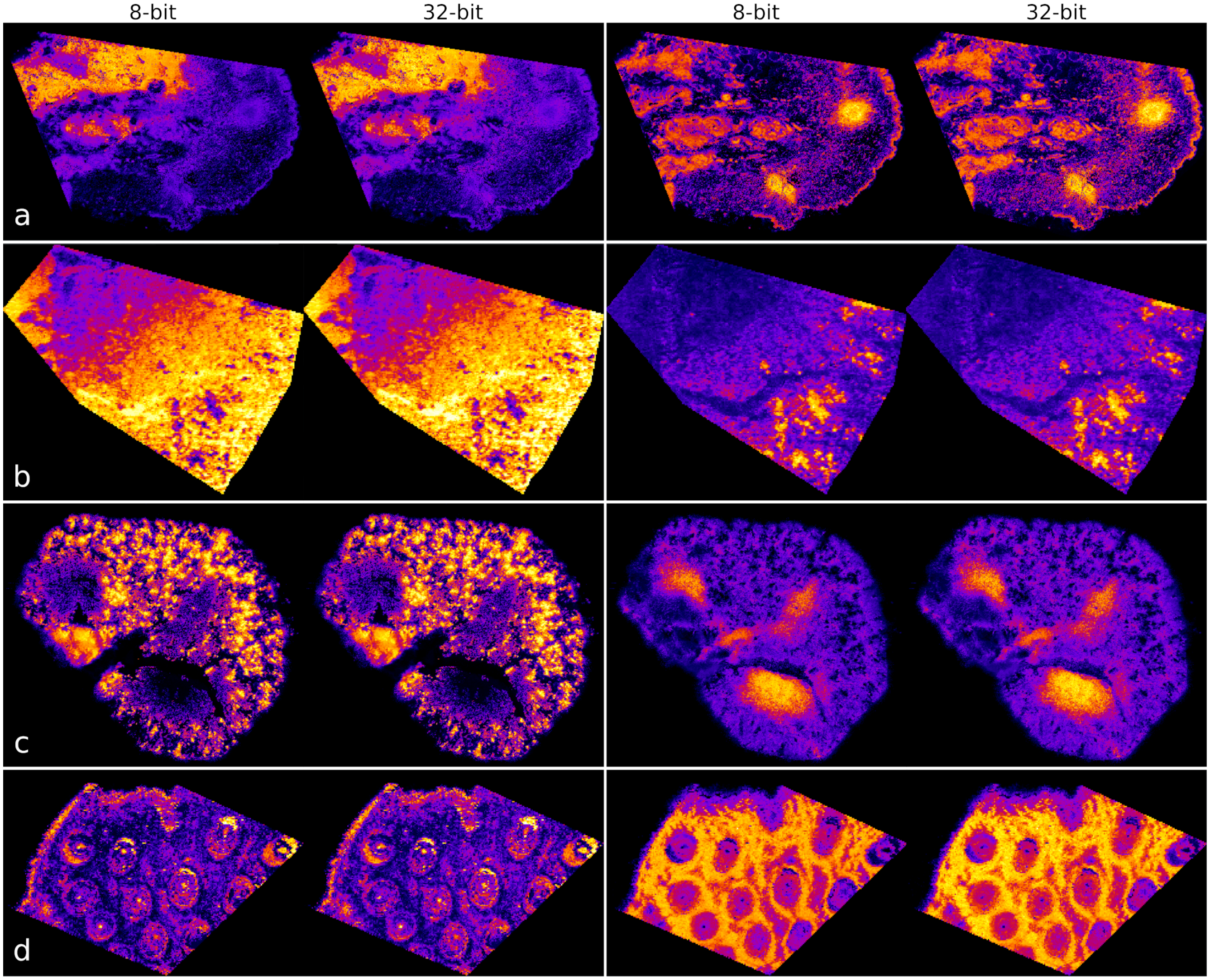

Figure 8. Comparison of 8-bit and 32-bit encoded QUIMBI visualization. Two example sets of QUIMBI visualizations with randomly chosen reference pixel positions are shown for row (a) data set I_s^t, row (b) data set I_k^t, row (c) data set I_k^O and row (d) data set I_v^O, respectively. The left column of each example set shows the QUIMBI visualizations based on the 8-bit encoding used in the main manuscript. The right column of each set shows the visualization on the same reference pixel position based on 32-bit encoding. There are no structural differences in the visualizations.

L Comparison of various dimension reduction methods

Figures 9 to 11 illustrate the comparison of four different dimension reduction methods for the purpose of matrix detection. It can be seen that the combination of PCA, NMF and tSVD with k-means providing a solid detection of the matrix. However, a look at the embeddings of these three methods shows that no grouping of data points can be identified manually. This has two major disadvantages: 1. One has to blindly trust in these methods. If the detect236580ion is not working as well as in this example, it can be very tedious to inspect the embedding manually as it provides no visual cues for potential clusters. 2. The automatic detection is not able to detect potential artifacts nor does the embedding provide visual clues for such spectra.

It can be seen that the use of simple clustering techniques like k-Means is not suited to detect clusters in embeddings with complex topology such as those generated by UMAP, which is the reason why we proposed a different method in Supplementary J. However, the embedding provides a lot of visual cues for potential clusters, which makes manual selection considerably easier.

In summary, all methods applied (PCA, NMF, tSVD and UMAP) deliver data embeddings that can be used to algorithmically determine point groups relating to matrix or artifact signals (for instance using machine learning). However, only the UMAP embedding provided an embedding plot with such a strong topological separation of matrix/artifact and sample clusters that this plot can be used as part of an interactive manual selection process, giving users full control over this sensitive data preparation step.

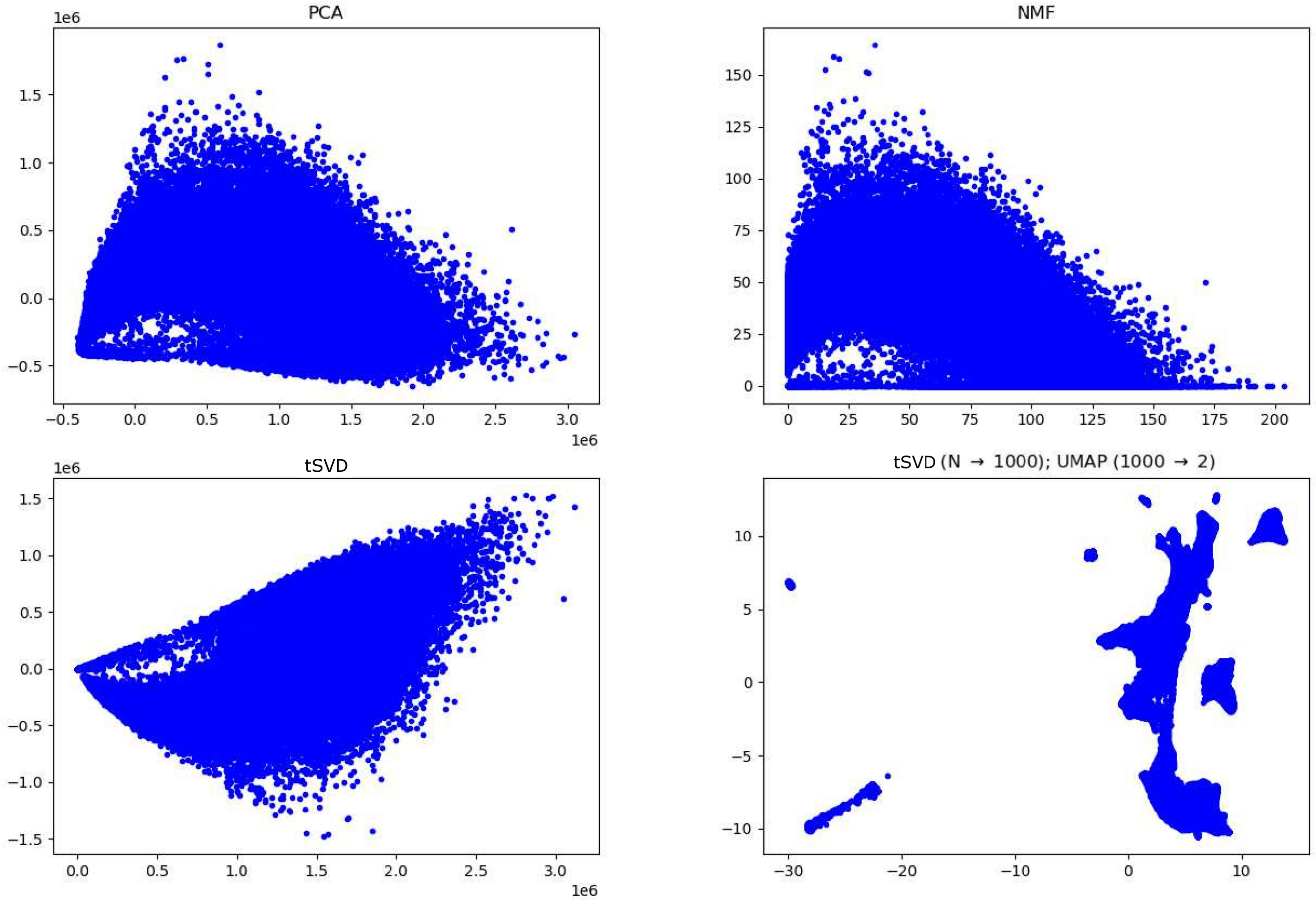

Figure 9. Comparison of four dimension reduction embeddings used on the mouse kidney data set.

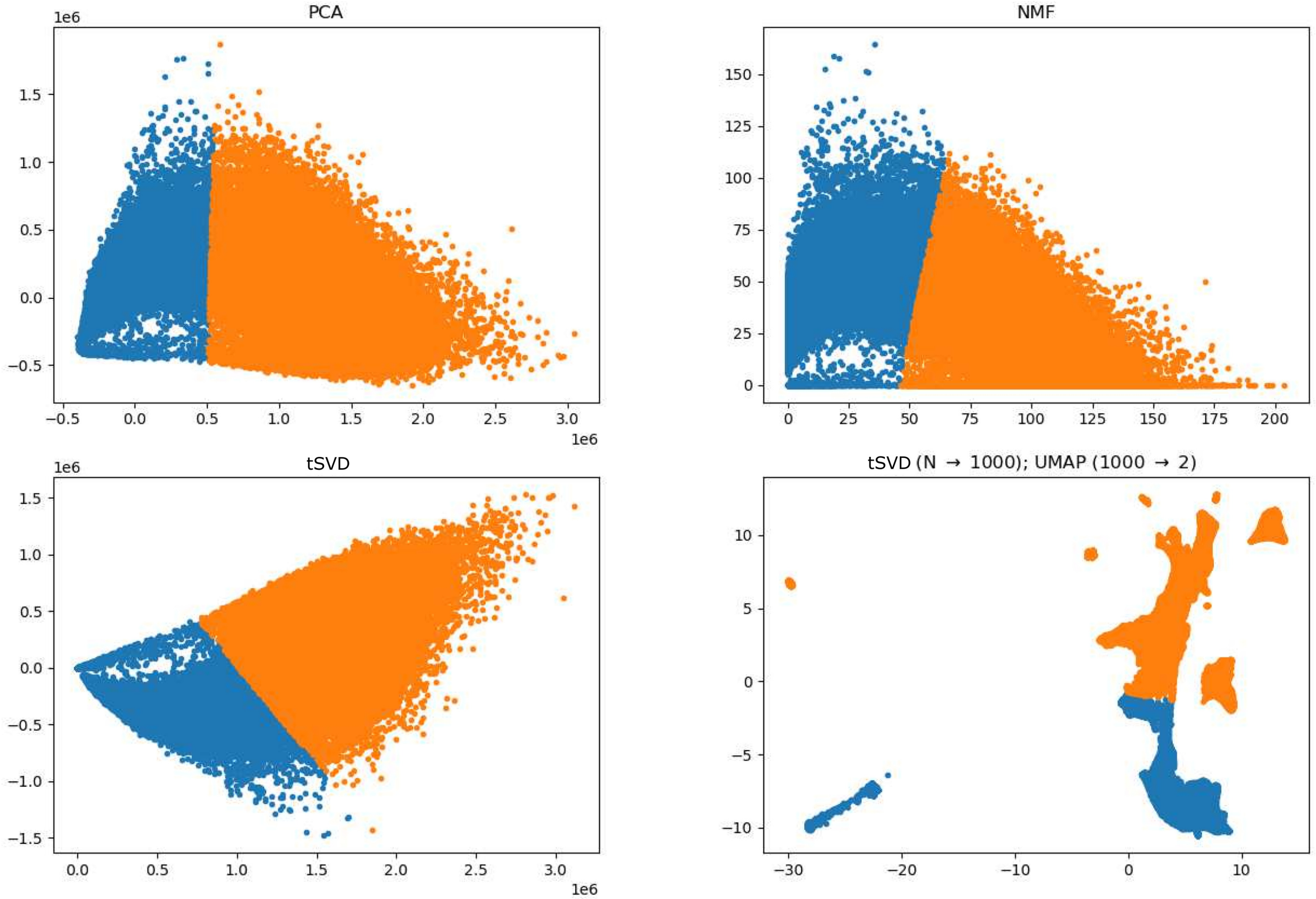

Figure 10. Comparison of four dimension reduction embeddings used on the mouse kidney data set. The two colors represent two different clusters detected with *k*-Means.

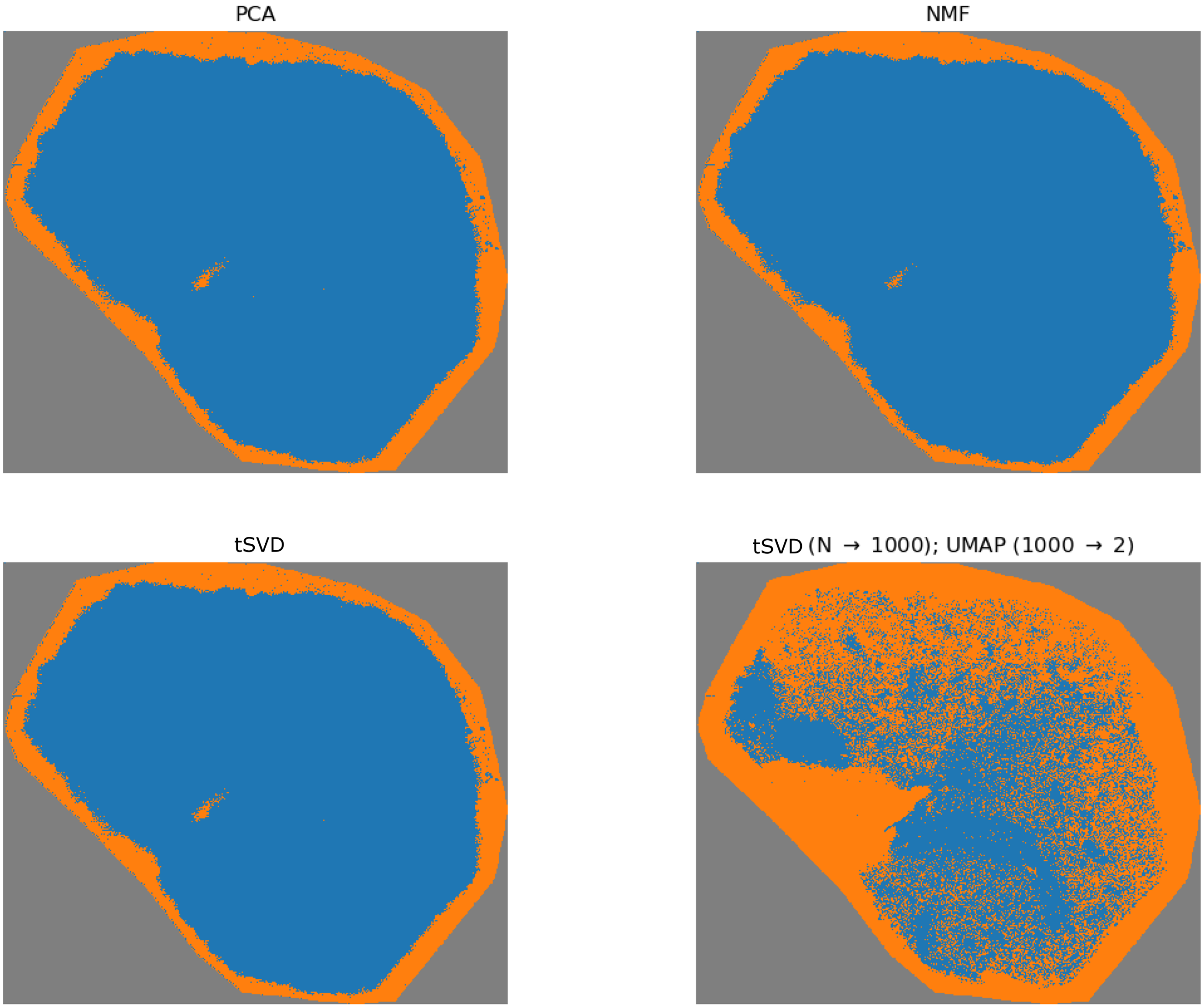

Figure 11. Segmentation maps resulting from the clusters found in Figure 10.

References

1. Halko, N., Martinsson, P.-G. & Tropp, J. A. Finding structure with randomness: Probabilistic algorithms for constructing approximate matrix decompositions. *SIAM review* **53**, 217–288 (2011).

2. Pedregosa, F. *et al.* Scikit-learn: Machine learning in Python. *J. Mach. Learn. Res.* **12**, 2825–2830 (2011).

3. scikit-learn. https://scikit-learn.org/stable/index.html. [Version: 0.20.2].

4. McInnes, L., Healy, J. & Melville, J. Umap: Uniform manifold approximation and projection for dimension reduction. *arXiv preprint arXiv:1802.03426* (2018).

5. Umap: Uniform manifold approximation and projection for dimension reduction. https://umap-learn.readthedocs.io/en/latest/. [Version: 0.3.9].

6. Van Der Walt, S., Colbert, S. C. & Varoquaux, G. The numpy array: a structure for efficient numerical computation. *Comput. Sci. & Eng.* **13**, 22 (2011).

7. Lux, M. *et al.* acdc–automated contamination detection and confidence estimation for single-cell genome data. *BMC bioinformatics* **17**, 543 (2016).
